# The receptor like kinase at *Rhg1*-a*/Rfs2* caused pleiotropic resistance to sudden death syndrome and soybean cyst nematode as a transgene by altering signaling responses

**DOI:** 10.1186/1471-2164-13-368

**Published:** 2012-08-02

**Authors:** Ali Srour, Ahmed J Afzal, Laureen Blahut-Beatty, Naghmeh Hemmati, Daina H Simmonds, Wenbin Li, Miao Liu, Christopher D Town, Hemlata Sharma, Prakash Arelli, David A Lightfoot

**Affiliations:** 1 Department of Molecular Biology, Microbiology and Biochemistry, Southern Illinois University at Carbondale, Carbondale, IL 62901, USA; 2 Department of Plant Soil and Agricultural Systems, Southern Illinois University at Carbondale, Carbondale, IL 62901-4415, USA; 3 Department of Horticulture and Crop Science, Ohio State University, 2021 Coffey Rd, Columbus, OH 43210, USA; 4 Agriculture and Agri-Food Canada, Building 21, 960 Carling Ave, Ottawa, ON K1A 0C6, USA; 5 Key Laboratory of Soybean Biology in the Chinese Ministry of Education, Harbin University, Harbin, China; 6 JCVI, Rockville, MD, USA; 7 Department of Plant Breeding & Genetics, Rajasthan College of Agriculture, MPUAT, Udaipur, India; 8 USDA, Crop Genetics Research Unit, Jackson, TN, USA; 9 Genomics Core Facility; Center for Excellence the Illinois Soybean Center, Southern Illinois University at Carbondale, Carbondale, IL 62901-4415, USA

**Keywords:** Segregation, Pleiotropy, *Rhg1/Rfs2*, Soybean, Resistance, Soybean cyst nematode (SCN), Sudden death syndrome (SDS), Insect herbivory

## Abstract

**Background:**

Soybean (*Glycine max* (L. Merr.)) resistance to any population of *Heterodera glycines* (I.), or *Fusarium virguliforme* (Akoi, O’Donnell, Homma & Lattanzi) required a functional allele at *Rhg1/Rfs2*. *H. glycines*, the soybean cyst nematode (SCN) was an ancient, endemic, pest of soybean whereas *F. virguliforme* causal agent of sudden death syndrome (SDS), was a recent, regional, pest. This study examined the role of a receptor like kinase (RLK) *GmRLK18-1* (gene model *Glyma_18_02680* at 1,071 kbp on chromosome 18 of the genome sequence) within the *Rhg1/Rfs2* locus in causing resistance to SCN and SDS.

**Results:**

A BAC (B73p06) encompassing the *Rhg1/Rfs2* locus was sequenced from a resistant cultivar and compared to the sequences of two susceptible cultivars from which 800 SNPs were found. Sequence alignments inferred that the resistance allele was an introgressed region of about 59 kbp at the center of which the *GmRLK18-1* was the most polymorphic gene and encoded protein. Analyses were made of plants that were either heterozygous at, or transgenic (and so hemizygous at a new location) with, the resistance allele of *GmRLK18-1*. Those plants infested with either *H. glycines* or *F. virguliforme* showed that the allele for resistance was dominant. In the absence of *Rhg4* the GmRLK18-1 was sufficient to confer nearly complete resistance to both root and leaf symptoms of SDS caused by *F. virguliforme* and provided partial resistance to three different populations of nematodes (mature female cysts were reduced by 30–50%). In the presence of *Rhg4* the plants with the transgene were nearly classed as fully resistant to SCN (females reduced to 11% of the susceptible control) as well as SDS. A reduction in the rate of early seedling root development was also shown to be caused by the resistance allele of the *GmRLK18-1*. Field trials of transgenic plants showed an increase in foliar susceptibility to insect herbivory.

**Conclusions:**

The inference that soybean has adapted part of an existing pathogen recognition and defense cascade (*H.glycines*; SCN and insect herbivory) to a new pathogen (*F. virguliforme;* SDS) has broad implications for crop improvement. Stable resistance to many pathogens might be achieved by manipulation the genes encoding a small number of pathogen recognition proteins.

## Background

*Fusarium virguliforme* (Akoi, O’Donnell, Homma & Lattanzi)*,* causal agent of soybean (*Glycine max* L. Merr.) sudden death syndrome (SDS), first caused a significant disease loss in 1987 [1]. *F. virguliforme* was not prevalent in Asia by 2011 but had spread quickly across the Americas from about 1980–2011. SDS has become a major pest problem for soybean growers and breeders in the Americas [2]. The origins of the disease remain unclear but *F. virguliforme* may be a new pathogen of soybean since no complete resistance has been reported.

*F. virguliforme*, like many plant pathogenic Fusaria, were facultative hemi-biotrophic pathogens of plant roots with many host species [1,3,4]. However, only soybean among known hosts showed the leaf scorch when infected by *F. virguliforme.* Soybean cultivars showed a wide range of susceptibility to both leaf scorch and root rot suggesting cultivar-specific partial resistance existed [5]*F. virguliforme* appeared to be a clonal pathogen [3,6]. There were some variations in aggressiveness among field isolates and maintained strains but there were no races reported, by 2011.

Soybean resistance to SDS was multi-geneic and had two components; a partial resistance to root infection and rot caused directly at the site of infection by the fungus; and a partial resistance to leaf scorch caused indirectly by translocated fungal toxins [5,7,8]. The bases of resistance might include resistances to one or more toxins [9]; and both local and systemic resistances following pathogen recognition [10-12].

*Heterodera glycines* I., the soybean cyst nematode (SCN) was probably an ancient pest of soybean since complete resistances to some Hg Types of SCN was found in about 1% of pre-domesticated and early domesticated Plant Introductions (PIs) [13]. Interestingly most of these PIs were also partially resistant to SDS [14]. One locus, the *Rfs2/Rhg1* locus on chromosome 18, was shown to underlie coinheritance of resistance to SDS in the roots and also reduce root infestation by SCN [10,11,14-17]. Fine map development did not resolve *Rfs2* from *Rhg1* suggesting the underlying gene(s) were either very closely linked or pleiotropic [11,16].

*Rfs2* underlay partial resistance to the spread of root infections by *F. virguliforme *[5,7,11]. The site of infection did not rot as rapidly when this allele was present and rates of root growth nearly equal to non-infested plants were maintained. Toxin translocation to leaves appeared reduced because leaf scorches did not develop or were less severe. The sudden plant death characteristic of SDS was manifested as both early senescence and an unusual abscission, basal to the leaflets instead of the petiole. Neither occurred if the *Rfs2* allele was present.

Equally the *Rhg1* locus underlay partial resistance to SCN [13,18]. *H.glycines*, like many plant parasitic nematodes, were obligate endoparasites of plant roots. Like *F. virguliformeH. glycines* has many alternate hosts. Over the past 50 years, the number of Hg Types (ex. races) has expanded from 4 in the 1960’s to 16–20 [19,20]. However, the *Rhg1* locus was constant, being required for partial resistance to all Hg Types in most PIs and cultivars. Full resistance to SCN required 1–4 loci in addition to *Rhg1*, the number depending on the nature of the cyst population parasitizing the roots [17,21-25]. Genetic diversity was found among SCN isolates, even inbred cyst populations like PA3, Hg type 0 [13,26]. Further, variation among the host plant roots response to SCN has been associated with temperature [27] such that environmental conditions must be rigorously controlled during assays [13,16].

The resistance or susceptible interaction(s) between the nematode and soybean affected by *Rhg1* was not induced until females stopped moving through the roots and established a feeding site comprising several giant cells [28-30]. Full resistance to SCN, based on the combined action of the genes at *Rhg1* and one or more additional *Rhg* loci, was manifest as; cell wall appositions to surround the feeding site; failure to supply the feeding site a tracheary element; and a necrosis as the feeding site develops. If the resistance allele at *Rhg1* was present normal rates of root growth were slightly depressed but the above ground stunting, yellowing and early senescence did not occur.

Inheritance of resistance to SCN was first reported in the PI ‘Peking’ [13]. Three recessive loci (*rhg1rhg3*) and a dominant locus (*Rhg4*) were assigned gene names by parsimony though other dominance models were equally likely. The Peking derived resistance alleles of *rhg1* and *Rhg4* were introgressed into the cv. ‘Forrest’ [31-33]. In crosses based on Forrest and with SCN isolate PA3 the *rhg1-a* was shown to be codominant with the susceptibility allele of ‘Essex’ (*rhg1*-e) and alone capable of providing partial resistance. Consequently, the Forrest, ‘Hartwig’, Peking and ‘PI 437654’ allele was renamed to *Rhg1-a* ([17] and hereafter) by the Soybean Genetics Committee.

The *Rhg1* locus was located to a sub-telomeric region of the soybean chromosome 18 (molecular linkage group G; Lg G) by many studies [17,21,23-25]. All of these segregating populations that were later tested with *F. virguliforme* also had an *Rfs2-*like activity against SDS [8,15,34]. However, an *Rhg1*-like locus was found at other locations in a few SCN resistant PIs, including Lg B1 (chromosome 11) [22], mid LgG [35,36] and Lg B2 (chromosome 14) [37,38]. The effects of the *Rhg1*-like loci found at other locations than chromosome 18 on resistance to SDS were not reported by 2012.

It has been shown the resistance allele of *Rhg1*-a*/Rfs2* (from Peking) was associated with reduced seed yield when SCN is not present in the fields [39-41]. That phenomenon might be related to delayed seedling development and stand formation [42]. How the root reduction contributes to resistance may involve a locus on chromosome 7 (LG M) where *Rzd*, an interacting allele needed for resistance to zygote death, was strictly co-inherited in phase with *Rhg1/Rfs2*-a [21]. Therefore, effects on development were predicted for the gene(s) underlying *Rhg1-a/Rfs2-a *[16,43-47].

Fine scale genetic maps and BAC based genomic analysis identified a 42 kbp region encompassed in BAC B73p06 as the *Rhg1/Rfs2* locus [16,45,48]. The region encoded an RLK (*Glyma18g02680* named *GmRLK18-1* hereafter), a variant laccase (*Glyma18g02690*) and a predicted Na/H ion antiporter (*Glyma18g0270*). The *GmRLK18-1* and *Gmlaccase18-1* were expressed in roots, shoots and flowers but not nodules or seeds. The antiporter was not expressed in any organ tested in Essex or Forrest. However, it might be expressed in flowers and seed at very low abundance [49]. Many nucleotide differences were found in the region encompassing the *GmRLK18-1* and *Gmlaccase18-1* genes from fragments of sequences from the resistant allele in Forrest compared to susceptible genotypes ‘Asgrow 3244’ and ‘Williams 82’ [16,29,48,50,51]. However, the *GmRLK18-1* was considered the most likely candidate gene underlying *Rhg1*-a based on fine maps and association analyses.

RLKs are part of the eukaryotic tumor necrosis factor beta receptor super-family [44,52]. In plants they represent a major class of resistance genes and also a major class of developmental regulators. The *GmRLK18-1* gene at *Rhg1/Rfs2* had nine alleles recognized among PIs and cultivars [16,44-48,50]. Of those four alleles were associated with partial resistance to SCN and five with susceptibility. The second most important resistance allele was from PI88788 and named *rhg1*-b because it was recessive and discovered second [53]. However, a small scale experiment with RNAi to the RLK (named *GmRLK18-1*-b) at the *rhg1*-b in transgenic hairy roots did not show a large effect on cyst numbers [53]. Either dominance or incomplete inhibition of the protein might have occurred. Here, additional experiments were undertaken with the *GmRLK18-1* at *Rhg1*-a in stable transformed soybean lines. Here, a molecular basis for resistance to SDS and SCN was inferred from functional analyses of the *Rhg1/Rfs2*-a locus in near isogenic lines (NILs) and the Forrest allele of the *GmRLK18-1* gene in transgenic plants.

## Results

### Allelic variations at the *Rhg1/Rfs2* locus

**Figure 1:**
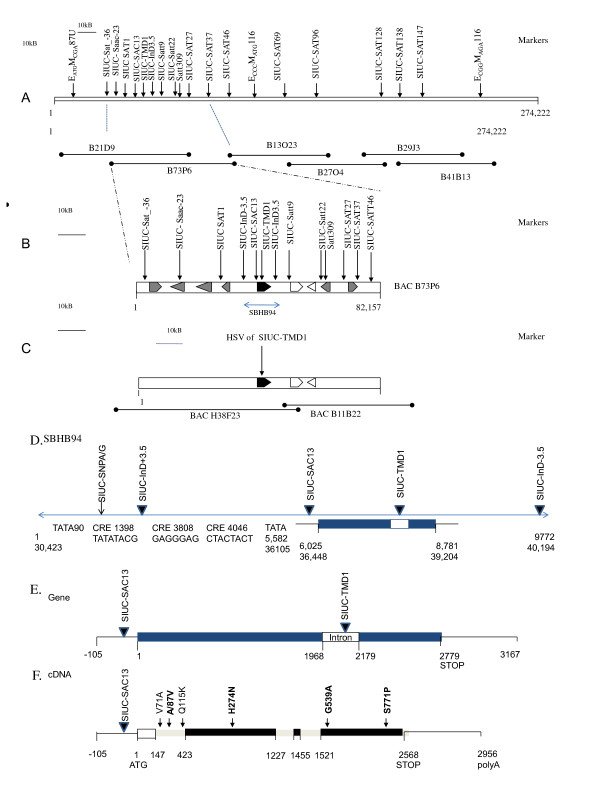
**Marker map of the genomic region around *Rhg1/Rfs2* and the most identical paralog (homeolog) of *Rhg1/Rfs2* with locus, BAC and gene ideograms.** Panel **A** shows the marker map of the genomic region around Rhg1/Rfs2 (Lg G; chromosome 18) with locus ideograms. Sequence coordinates were from the susceptible cultivar Asgrow 3244 [16,49]. The *GmRLK18-1* gene encoding the RLK was shown as a black block arrow. The genes encoding the laccase and antiporter were shown as opposite white block arrows. All other genes were shown as grey block arrows. Locations of overlapping BAC clones B73p06 and B21d09 that both encoded the *GmRLK18-1* at the *Rhg1/Rfs2* locus (Lg G; chromosome 18) were shown below the ideogram. Panel **B** shows the BAC clone B73p06 that encoded the *Rhg1/Rfs2*-a locus. The gene encoding the RLK was shown as a black block arrow. The genes encoding the laccase and antiporter were shown as opposite white block arrows. All other genes were shown as grey block arrows. The extent of the pSBHB94 (HQ008939), the 9.772 kbp subclone from BAC B21d09 that was used for soybean transformations was shown as a blue arrow. The plasmid pSBHB94 encompassed from 30,423- 40–194 bp. Sequence coordinates were from the complete sequence of the BAC derived from resistant cultivar Forrest (HQ008938). Panel **C** showed a syntenic homeolog of *Rhg1*-a /*Rfs2/* found in the sequence of BAC H38F23 from Lg B1 (chromosome 11). The homeolog of the gene encoding the RLK was show as a black block arrow. The homeologs of the genes encoding the laccase and antiporter were shown as opposite white block arrows. All other syntenic genes were shown as grey block arrows. The marker TMD1 amplified a fragment from *Rhg1*-a /*Rfs2/Rhg1*-a*/Rfs2* of 303+ 15 bp (resistant allele was the smaller) and of 362 bp from a syntenic homeolog of *Rhg1*-a /*Rfs2Rfs2/* found in the sequence of BAC H38f23 from Lg B1 (chromosome 11). Sequence coordinates were from the complete sequence of the BAC derived from resistant cultivar Forrest (HQ008940). Panel **D** shows an ideogram of markers, the *GmRLK18-1* gene and gene features found in the insert of plasmid pSBHB94 that encompassed from 30,423- 40,194 bp of B73p06. Panel **E** shows the position of markers in the transcribed region of the *GmRLK18-1* gene. Panel **F** shows the position of amino acid substitutions in the protein encoded by the cDNA.

The Forrest (resistance) allele of the *Rhg1/Rfs2* locus was analyzed by sequencing the entire BAC B73p06 to 8 fold redundancy (Figure 1; GenBank HQ008938). The BAC sequence was compared to the sequences of (susceptible) Williams 82 whole genome shotgun sequence [54] and a BAC contig developed from (susceptible) ‘Asgrow 3244’ [50] from the same region. The BAC insert encompassed 82,157 bp that was predicted to encode nine genes comprised of 57 exons (2–8 per gene; Table 1). The *GmRLK18-1* was gene 5, the laccase gene 6 and the antiporter gene 7. Sequences of the resistant and susceptible alleles were colinear with no large (more than 100 bp) insertions, deletions or inversions. Across the entire BAC there were 800 SNPs between Forrest and the sequences of alleles for susceptibility. SNPs were found in the promoter and enhancer regions of all 9 genes. However there were only 31 SNPs within genes. Only 11 of the 31 caused amino acid changes. There were just 6 of the 9 proteins changed by those 11 SNPs.

**Table 1:** **Genes predicted from DNA sequence within the *Rhg1* -a/ *Rfs2* locus encoded by BAC 73p06 (81, 157 bp) from Forrest (resistant) and sequence contig from A3244 (susceptible; AX196295)**

**Gene #**	**Annotation**	**Forrest**	**A3244**	**EST**
Marker	SIUC_Sat_−35 (35 bp)	1,770	11,870	na
**1 (5)**	**NADP redox coenzyme-like**	**5,959–7,947**	**16,234–18,215**	**AW185583**
**2 (6)**	**Predicted protein**	**14,800–10,888**	**25,760–21,294**	**BG550903**
*Marker*	*SIUC_Scaa-23 (19 bp)*	*13,800*	*23,900*	*na*
**3 (7)**	**Predicted protein**	**23,795–18,782**	**34,265–28,983**	**TC63131**
Marker	SIUC_Sat_001 (2 bp)	24,500	34,600	na
**4 (8)**	**Predicted protein**	**30,076–28,429**	**40,482–38,327**	**na**
Marker	SIUC_InD + 3.5 (53 bp)	33,900	44,000	na
*Marker*	*SIUC_Sca_005/Sac_013 (4 bp)*	*36,400*	*46,700*	*na*
**5 (9)**	**Receptor like kinase ( *Rhg1* -a/ *Rfs2* )**	**36,448–39,204**	**46,891–49,573**	AF506517*
*Marker*	*SIUC-SattTMD1(19 bp)*	*38,500*	*48,500*	*na*
Marker	SIUC_Indel-3.5 (22 bp)	40,150	50,000	na
Marker	STS 10893 (12 bp)	43,800	54,000	GF097715
Marker	SIUC-Satt9.0 (17 bp)	45,250	55,000	na
Marker	SIUC-Satt9.5 (21 bp)	45,500	55,000	na
**6 (10)**	**Variant diphenol oxidase**	**47,930–52,465**	**58,247–62,782**	AY113187*
Marker	SIUC-SNP_A/G	56,320	66,637	na
**7 (11)**	**Na/H antiporter-like 1**	**57,661–55,772**	**67,540–64,896**	**na**
Marker	SIUC-Satt22/Indel22 (20 bp)	58,500	58,500	na
*Marker*	*BARC-Satt309 (17 bp)*	*61,100*	*71,400*	*na*
**8 (12)**	**Na/H antiporter-like 2**	**64,165–60,904**	**74,602–69,934**	AW279576*
**9 (13)**	**DNA helicase-like 1**	**64,996–73,760**	**75,245–75,418**	**na**
*Marker*	*SIUC-Sat_027(3 bp)*	*65,570*	*75,881*	*na*
**10 (14)**	**DNA helicase-like 2**	**70,601–74,056**	**80,601–84,056**	**BF425110**
Marker	SIUC- ATG4	74,150	84,400	na
Marker	SIUC- Sat_37	74,250	84,500	na
Marker	Minisat 2/SIUC-Sctt39 (45 bp)	75,800	85,900	na
Marker	SIUC-Sat_40 (38 bp)	79,500	89,600	na

Shown are; the gene numbers in the larger sequenced interval; the sequence coordinates from the predicted 5′ translation start to 3′ translation stop; and the presence of ESTs in Genbank (May 2012) with homology to the predicted genes. Marker coordinates are listed. Intra-geneic markers were italicized. Genes were bold face. Marker positions are rounded to nearest 10 bp except for SNP markers which were exact. The * indicated the aligned EST and mRNA were from soybean, *Glycine max.* The plasmid pSBHB94 encompassed from 30,423- 40–194 bp in BAC 73p06.

There was evidence for a large and highly polymorphic region within the BAC (743 SNPs in 59 Kbp; from 1,500–60,500 bp). A highly polymorphic region was expected to be a characteristic of the region introgressed into Forrest from Peking. Equally, relatively monomorphic regions were common when comparing sequences of US cultivars [54,55]. In fact, the three alleles (Forrest, Williams 82 and A3244) were nearly identical outside the 59 kbp central region. For example the region that encompassed gene 10 had just 5 SNPs across 18.25 kbp among the 3 alleles and no alloproteins.

The RLK contained 2 of the 6 SNPs in the BAC predicted to cause amino acid changes (Ruben et al. 2006; Afzal et al. 2012) when comparing Forrest allele to Williams 82 and Asgrow 3244. However, it contained 7 synonymous SNPs. In comparison the laccase contained 3 SNPs predicted to cause amino acid changes and only one that was synonymous (Figure 1). The antiporter alloproteins contained one non-synonymous and two synonymous SNPs.

The greater number of differences between alleles of the *GmRLK18-1* and the other candidate genes was taken as evidence this should be the first gene tested in transgenic plants. The RLK was subcloned on a 9,772 bp insert for plant transformation (Figure 1D) that contained 6 Kbp of 5′ sequence, 2.7 kbp of geneic sequence and 1 kbp of 3′sequence. The 5′ sequence extended to the TATA box of the neighboring gene (#4). The region contained 7 potential cis-regulatory elements (CREs) of 8 bp that were identical to motifs found in the 5′ regions hundreds of plant genes. The four closest to the RLK are shown in Figure 1D. Only the most distant was polymorphic between cultivars. The Forrest allele of this CRE was the identical to sequences found 5′ to 1,091 rice genes and 108 Arabidopsis genes. There was a 53 bp deletion from Forrest (at 33,942 bp) compared to Essex, Williams 82 and Asgrow 3244 in the enhancer region of the gene that was named SIUC-indel-(plus) + 3.5. There was also a 22 bp deletion 3′ to the gene at (at 40,215 bp) named SIUC-indel (minus)-3.5. This deletion was within a complex microsatellite repeat mainly composed of AAAG motifs. Neither deletion appeared to encompass any previously characterized CREs. In the intergeneic region between RLK and laccase were four polymorphic small indels or microsatellite-like polymorphisms.

### Syntenic paralogs of the *Rhg1/Rfs2 locus*

The soybean genome was hypothesized to be the product of a diploidized tetraploid [54,55]. Therefore, a detailed molecular analysis of the *Rhg1/Rfs2* locus required that paralogs and syntenic gene clusters be identified. The most identical paralog (the homeolog) was found on BAC H38f23 (Additional file 1: Figure S1) that was from Lg B1 (chromosome 11) in a region where loci with functions similar to *Rhg1/Rfs2* were mapped [22,36,37]. Significantly, the sequence of H38f23 contained a complete set of syntenic genes for a second *Rhg1/Rfs2* locus and surrounding genes (an RLK, laccase, both antiporters, the kinase and the helicase; *Glyma11g35710- Glyma11g35660*; Figure 1; HQ008940). Interrogation of Soybase showed the transcript abundance patterns for the syntenic RLK pair and laccase pair were both abundant in roots as reported in the RNA sequence atlas generated from a Williams 82 sister line derived NIL (cultivar ‘P-C609-45-2-2’ a BC5F5 plant derived from *G. soja* (‘PI 468916’) backcrossed into *G. max* (‘A81-356022’) [49]. The laccase transcripts were 15 fold more than either of the RLK transcripts in roots. Though it must be noted this genotype was either the *rhg1*-d or *Rhg1*-e allele.

Comparing the syntenic BACs the DNA sequence identity was high (~97% in geneic regions; Additional file 1: Figure S1E; Additional file 2: Table S1). At the RLKs, *GmRLK18-1* and *GmRLK11-1*, with intron boundaries as described by [16] and [25], the amino acid identity was 93% overall with 94% in the LRR, 93% in the trans-membrane domain and 97% in the kinase domain (Additional file 1: Table S1). The N terminal signal peptide was most divergent (11 changes in 50 amino acids). The laccase and the antiporter also showed 85–96% amino acid identity with their syntenic paralogs.

*GmRLK11-1*’s amino acid sequence was less identical to *GmRLK18-1* than the alleles (98–99%) of the gene. However, four of the six residues that differ among alloproteins of *GmRLK18-1* were identical to the Forrest allele in the homeologous protein GmRLK11-1-a. At the other 2 residues the changes were identical to the susceptible alleles of Essex and Williams 82. From sequence comparison and transcript abundances it appears likely the paralogs *GmRLK18-1* and *GmRLK11-1* would share functions and should hetero-dimerize [47].

### Allele and paralog discrimination in NILs and transgenic plants

**Figure 2:**
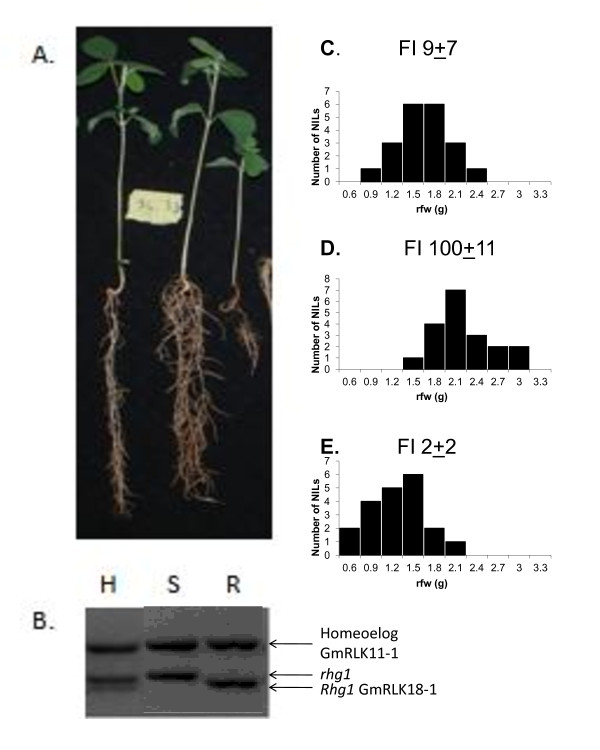
**The *Rhg1/Rfs2* genomic region altered root development.** Panel **A**; soybean NILs at 2 weeks pre-SCN inoculation show different root morphologies, *Rhg1/Rfs2* inhibited germination and early root growth. Panel **B**; the root morphologies co-segregated with the allele of *GmRLK18-1* at the *Rhg1/Rfs2* locus as shown by the 3 bands generated by intron located marker TMD1 (arrowed). R indicated the resistant allele from Forrest; **S** the susceptible allele from Essex; and H indicated the heterozygous state for the *Rhg1/Rfs2* locus. Panel **C** showed the distribution of root mass (rfw was root fresh weight) among 20 heterozygous plants. Panel **D** showed the distribution of root mass among 20 susceptible plants. Panel E showed the distribution of root mass among 20 resistant plants.

Since paralogs with homeolog sequence variants (HSVs) appeared to exist for each gene in the cluster, it was important to distinguish alleles precisely and separately from their most similar HSVs. In the NIL population RLK alleles and HSVs were distinguished using both the SIUC-TMD1 marker (Figure 2) and SNP Ala87Val from the LRR region by Taqman (Additional file 3: Figure S2; Additional file 4: Table S2). In genomic DNA TMD1 distinguished both the Peking and Peking-like allele of *GmRLK18-1* from the other 7 alleles and the homeologous alleles of *GmRLK11-1*. To distinguish Forrest from both Essex and X5 the SNP probe to Ala87Val was again used for both genomic DNA and cDNA.

### Dominant, recessive or co-dominant nature of *Rhg1/Rfs2* in NILs

In the NIL line 34–33 that was heterogenous at the *Rhg1/Rfs2* locus, about 12% of plants (4/34) were still heterozygous by the F5:7:13 (thirteen generations after the original cross and single seed selection at F5; NIL selection at F7; Figure 2). Those plants were heterozygous at the TMD1 marker because they had not yet been fixed by recombination. The frequency of heterozygous plants among F5:7:13 generation seed was surprisingly high (4/34 or 12%) compared to the expected frequencies at the F5 (6.25%), F7 (1.56%) and F13 (0.025%) generations. Even calculating the expected heterozygous plant frequency starting from the F5 the occurrence was higher than expected (1/256 or 0.39%). The existence of these plants suggested that fixation by re-assortment was selected against in viable zygotes (or gametes) or that the heterozygous state was under positive selection. The remaining 30 plants (88%) were fixed equally to Forrest or Essex alleles (15/34 or 44% each).

The cyst scores for all plants in the NIL population corresponded with the respective alleles at the *Rhg1/Rfs2* locus so plants with resistance alleles had female indices (FIs) less than 8% and plants with susceptibility alleles had FIs greater than 84% (Figure 2). For the four heterozygous plants, polymorphic at TMD1, the cyst scores ranged from 5–12% which corresponded to those for resistant or moderately resistant plants. Therefore, the *Rhg1/Rfs2* locus was dominant in this set of NILs infested with HgType 0 population JB3. However, heterozygous NILs were reported co-dominant with HgType 0 population PA3 in earlier tests where FIs ranged from 5–40% [23]. Co-dominant and recessive roles of plant disease resistance loci were previously associated with factors needed by the pest for successful parasitism [56,57]. The discrepancies in dominance among different populations may be associated with the genetic background in which the gene resides or may result from interactions among genes at the *Rhg1/Rfs2* locus and/or modifier genes at other loci [21].

### Inhibition of root growth by alleles of *Rhg1/Rfs2* in the NILs

When counting the cysts with prior knowledge of the allele at *Rhg1/Rfs2* in the standard assay of SCN it was noted that root mass and vigor appeared to differ among genotypes. Measurement of root masses showed a significant difference (P < 0.0015) among NILs that were associated with the allele at *Rhg1/Rfs2* (Figure 2). Across several experiments, both NILs that were susceptible and NILs that segregated most susceptible lines had higher root masses (mean 2.24 ± 0.19 g) than their SCN resistant (1.22 ± 0.22 g) or heterozygous (1.79 ± 0.18 g) counterparts. This phenomenon might underlie the global association of resistance to SCN with low seed germination, seedling vigor and ultimately seed yield noted previously [40-42]. In addition, the lower root growth might provide an avoidance mechanism that is part of a broad resistance effective against all Hg Types.

The distribution of recombination events found previously among the six Hg Type 0 susceptible PIs [16] suggested that the action of *Rhg1/Rfs2* required elements to the 3′ side of the RLK intron or in the C terminal portion of the protein. One of the 2 amino acid substitutions found in the intracellular kinase of the complete RLK (Gly539Ala and Ser770Pro) may be key to transducing intracellular signaling leading to the resistance response [44]. Some mutations in the kinases of other plant RLKs are known to be lethal [58-60]. Therefore, it may be the kinase at the *Rhg1/Rfs2* locus that underlies restricted root growth in resistant genotypes. The effect may be direct or occur after protein-protein interaction(s). The signal transduced is likely to result in a negative reponse since the kinase domain lacks the RD amino acid motif needed for ATP binding [44] and so protein phosphorylation.

### Transcripts of the *GmRLK18-1* alleles at *Rhg1/Rfs2* were found in soybean roots

**Figure 3:**
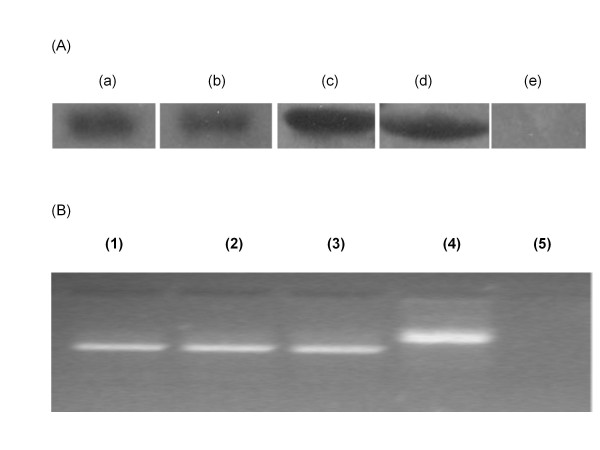
**Expression of Forrest (−a) and Essex (−e) alleles of the *GmRLK18-1* at *Rhg1/Rfs2*in non-transgenic soybean NIL roots.** Panel (**A**); Western hybridization using an anti-RHG1/RFS2 (*GmRLK18-1*) antibody (Afzal et al. 2007) from roots of; Forrest allele in ExF34-23 (a); Essex allele in ExF34-3(b); *E.coli* expressed proteins RHG1/RFS2-LRR-Shrt (c); RHG1/RFS2-LRR-Long (d); and RHG 4 (e). Panel (**B**); agarose gel electrophoresis of cDNA amplified using *Rhg1/Rfs2* LRR flanking primers from; non-infested NIL 34–23 (1); SCN infested NIL 34–23 (2); SCN infested NIL 34–3 (3); and Forrest genomic DNA (4). Negative control without template is shown in lane 5.

Expression judged by examination of EST libraries in silico, cDNA libraries by hybridization, RNA sequencing and mRNA populations assayed by qRT-PCR showed both transcripts encoded by *GmRLK18-1*-a and *GmRLK18-1*-e alleles (Figure 3) were present in both non-infested roots and SCN-infested roots, as reported in [16]. The transcript and protein abundances were not increased by more than 2 fold in either *H. glycines* or *F. virguliforme* infested plants compared to non-infested plants (Figure 3). Low transcript and protein abundances appeared to result from largely constitutive expression under the conditions and stages of development tested to date.

### Analyses of disease responses in plants transgenic with the resistance allele of the *GmRLK18-1* in the greenhouse

In order to test the hypothesis that *GmRLK18-1*-a underlay part of the activity of the *Rhg1/Rfs2*-a locus susceptible plants were made transgenic with this allele. Such plants had a resistance allele at a new location and a susceptibility allele (*GmRLK18-1*-a; *rhg1/rfs2*-e) on chromosome 18. Several primary transgenic lines were created by biolistics and fertile lines selected in two different cultivars (‘X5’ and ‘Westag 97’) for analyses of SDS and SCN responses.

**Figure 4:**
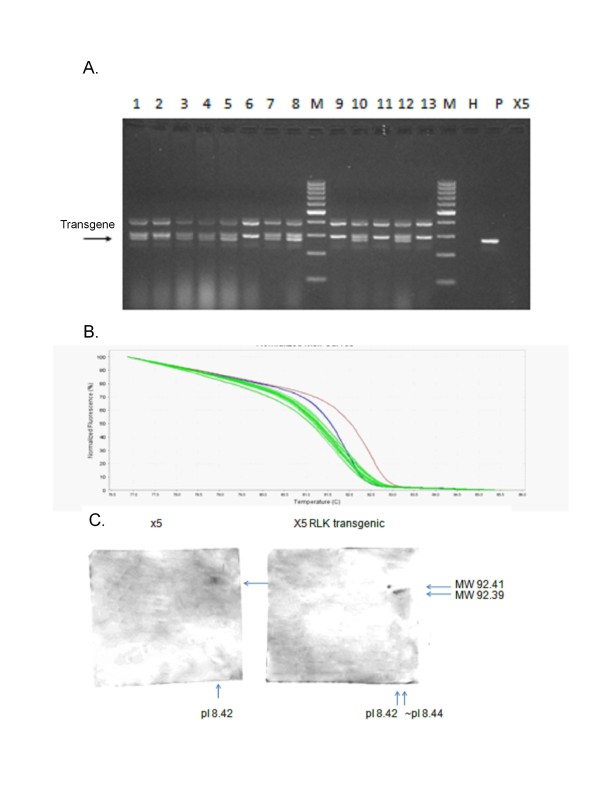
**Soybean transgenic plants expressed the mRNA and protein from the Forrest allele of *GmRLK18-1* , the RLK at the *Rfs2/Rhg1* locus.** Panel **A** shows PCR from leaf samples of progeny plants derived from a primary transgenic event 6B3-7D2(1) with TMD1 primers. Lanes contain transgenic plants 1 to 13. The arrow shows the double band for Gm18RLK-1-a positive sample at 314 bp for lines 1,3–5, 7,8,10 and 12. M was the marker; H was the no DNA (water) control; P was the *Rhg1* plasmid pSBHB94; X5 was the control plant. Panel **B** shows PCR from cDNA leaf samples of sixteen transgenic lines derived from event 6B3-7D2(1) with HRM primers. Green lines are from transgenic plants. Red melt curve was a resistant control blue line was a susceptible control. Panel **C** shows a Western of a 2D gel from roots of a transgenic plant probed with the anti-RLK peptide antibody. An alloprotein at pI 8.42 and 92.41 kDa was found in the non transgenic cv X5 but the presence of the Forrest alloprotein at pI 8.44 and and 92.39 kDa was found in transgenic plants derived from event 6B3-7D2(1) expressing *GmRLK18-1*-a. GmRLK18-1 was shown to be a very low abundance protein impossible to visualize without immune-staining.

In several transgenic lines from (T1-T3) used for SDS and SCN assays, lines were identified with the *GmRLK18-1*-a transgene in the homozygous state. In these plants the transgenes were both transcribed and translated. For example, in the progeny of fertile T0 line 6B3 the progeny T1 lines like 7D2 and sixteen T3 derived plants expressed the *GmRLK18-1*-a transgene as both mRNA and protein (Figure 4). Expression of the transgene alleles was equal to the endogenous gene alleles as judged by protein abundance. GmRLK1 transcripts and proteins were detected in about 50% of the progenies of the 6B3 primary transformants. In selfed lines expressing *GmRLK18-1*-a the transgene allele was detected as distinct mRNAs and alloproteins. Expression was directed by the native promoter and the enhancer elements contained on the proximal 6 kbp of the 9.772 kbp fragment of pSBHB94. That plasmid was sub-cloned from BAC B21d09 (Figure 1; HQ008939) during BAC sequencing.

**Figure 5:**
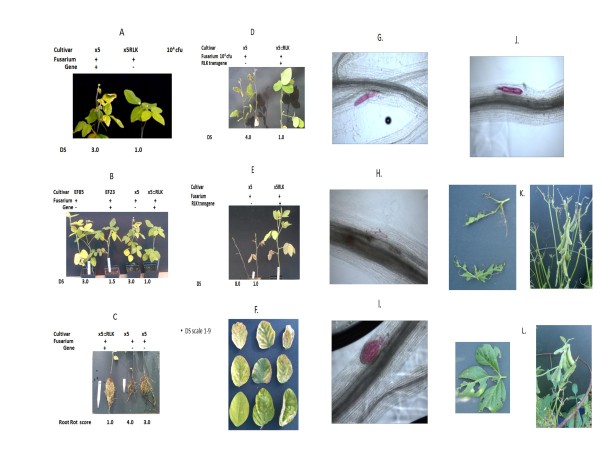
**Resistance to *Fusarium virguliforme* and partial resistance to *Heterodera glycines* caused by the Forrest allele of a receptor like kinase ( *GmRLK18-1* -a) found at the *Rfs2/Rhg1* locus as transgene in primary transgenic lines (cv ‘X5’).** Panels **A**-**H** show the SDS assays. *F. virguliforme* was used at 10^4^ cfu per cm^3^ of soil. The experiment was carried out on 3 separate occasions. Leaf scorch was scored as DS at 7, 14, 21, 28, 35, 42, 49 and 56 dai (days after infestation). Derived crossed lines (X5::*GmRLK18-1*-a x WENIL35 and cultivars X5:: *GmRLK18-1*-a x WENIL35xEF2) were included in runs 2 and 3. Panel **A** &**B** shows stable soybean transgenics with and without the 10 kbp *GmRLK18-1* (*Rhg1/Rfs2)* subclone at 21 dai. Panel **C** shows the *GmRLK18-1*-a transgene reduced root rot at 28 dai. Panel **D** shows leaf symptoms at 28 dai. Panel **E** shows plants at 56 dai where X5 is senescent with abscission of leaflets from erect petioles and X5::*GmRLK18-1*-a is still green and filling pods. Panel **F** shows selected leaflets at 28 dai with a 1–9 range in DS scores arranged in order of severity from bottom left to top right. Panels **G**-**J** show the SCN assays. Panel **G** shows SCN development by the RLK in X5 transgenics. Panel **H** shows SCN development by the *Rhg1-a* allele in resistant NIL 34–23. Panel **I** shows normal SCN development in the susceptible X5. Panel **J** shows normal SCN development in susceptible NIL 34–3. Panel **K** shows a *GmRLK18-1*-a transgenic plant of cultivar X5 that was defoliated by insect herbivory in the field. Panel **L** shows an non-transgenic X5 plant with much less leaf area loss.

In transgenic soybean plants *GmRLK18-1*-a allele provided resistance to both root infection and root rot by *Fusarium virguliforme.* That root resistance underlay a significant reduction in leaf symptoms and delay of the senescence caused by SDS (Figure 5; Table 2B). Resistance to SDS was effective throughout the life of the plants which flowered and set seed. The non-transgenic X5 plants proved to be highly susceptible to SDS and showed all the expected phenotypes of root rot and leaf scorch. The phenotypes among the susceptible plants included a gradual worsening of leaf scorch symptoms from 3.0 at 21 days after infection (dai) to 8.5 by 56 dai. Only at near maturity did the susceptible plants showed symptoms characteristic of SDS. For example, leaflet abscission occurred from the top of the petiole instead of the base of the petiole. However, the transgenic plants did not show early senescence or reduced pod set. Equally, the characteristic root symptoms of SDS were very reduced in the RLK transgenic plants. Symptoms reduced included the degree of root rot and browning of the root cortex. Senescence was delayed by 14 days in *GmRLK18-1* transgenics compared to controls and more pods were set in each of the three repeats of the experiment. Therefore, the *GmRLK18-1*-a provided a very high degree of resistance to the transgenic plants in roots and leaves and pods.

**Table 2:** Association of mean root growth in transgenic lines with pleiotropic resistance to two pests in two different greenhouse assays and insect herbivory in field tests

**A. Line::gene**	**SCN infested**	**Root mass (g)**	**Significant differences**	**Range (g)**	**n**	**SCN FI (%)**	
X5	No	1.05	a	0.81–1.44	15	0 ± 0.0	
Westag97	No	nd		nd	5	0 ± 0.0	
X5	Yes	0.98	a	0.73–1.31	15	100 ± 13	
X5::RLK	No	0.64	bc	0.57–0.74	15	0 ± 0.0	
X5::RLK	Yes	0.38	c	0.26–0.49	15	60 ± 11	
X5::RLK::*Rhg4*	Yes	nd		nd	2	11 ± 3	
X5::RLK::*rhg4*	Yes	nd		nd	3	38 ± 6	
Westag97	Yes	nd		nd	5	130 ± 11	
Westag97::RLK	Yes	nd		nd	5	12 ± 3	
B. Line::gene	Fungus infested	Root mass (g)	Significant differences	Range (g)	n	Root RS	Leaf DS
X5	No	7.80	a	4.90–8.78	15	1.0 ± 0.1	3.2 ± 0.7
X5	Yes	3.86	bc	3.08–4.80	15	4.5 ± 0.9	4.3 ± 0.8
X5::RLK	No	6.10	a	4.77–7.81	15	1.0 ± 0.1	1.0 ± 0.3
X5::RLK	Yes	4.64	b	4.00–6.22	15	1.5 ± 0.5	1.5 ± 0.4
Westag97	Yes	6.15	A	3.02–9.55	6	6.5 ± 1.9	6.3 ± 1.3
Westag97::RLK	Yes	12.2	B	3.55–24.0	6	0.5 ± 0.3	1.8 ± 0.5
C. Line::gene	Insect PI (%)	Mean shoot dry weight (g)		n	Leaf defoliation (%)	Seed No	weight
X5	22 + 13	47.2	a	4	33 ± 4.0	40 ± 9	11.6 ± 0.3
X5::RLK	59 + 15	18.8	b	4	90 ± 5.0	36 ± 8	11.2 ± 0.3

Part A shows SCN female index in greenhouse grown seedlings at 28 days after SCN infestations. Pots were watered daily with 100 ml. Female index (FI) was the mean percentage of cysts of Hg Type 0 found on five plants per repetition compared to a susceptible genotype Essex. Part B shows the effects of the transgene on resistance to *F. virguliforme*. in greenhouse grown seedlings at 28 days after infestations. Pots were saturated with water to the 5 cm level. Leaf scorch was recorded as the mean disease severity (DS) measured on a 1–9 scale found on five plants per experimental repeat. Root rot severity (IS) was measured on a 1–5 scale. Panel C shows the percent insect incidence, defoliation by herbivorous insects and the consequent loss of biomass at harvest as mean dry weight per plant for field grown plants with 4 replications across 2 years.

The results from X5 transgenics were comaperd to results from Westag97 transgenics in the SDS assay with infested plants (Table 2). Westag97 plant were bigger and more stress resistant than X5. However, the Westag plants transgenic with the RLK again proved more significantly more resistant to SDS caused by *F. virguliforme.* Therefore, the resistance was not transgenic event or cultivar specific.

In separate assays with plants from the same lines the resistance to SCN was partial in the *GmRLK18-1*-a transgenic plants (Table 2A) judged by female index (FI). Partial resistance was expected as *rhg4*-e was present and *Rhg4*-a absent. SCN FI was reduced by 30–50% across four experiments using three Hg Types (P < 0.01). Because the X5 selfed transgenic plants reported here had a susceptible allele at *Rhg4,* partial resistance was the expected outcome. The partial resistance was confirmed with another isolate of SCN at Harbin University (China). A cross was made to the RIL EF2 via a susceptible NIL WE1. EF2 was *rhg1*-e*/rfs2, rhg1*-e*/rfs2, Rhg4, Rhg4*. The progeny segregated for the RLK and so were *rhg1*-e*/rfs2, rhg1*-e*/rfs2, Rhg4, rhg4::*RLK18-1 or *rhg1*-e*/rfs2, rhg1*-e*/rfs2, Rhg4, rhg4* with no transgene. Plants with the RLK had a 20% lower FI and were almost in the resistant class (FI < 10; Table 2). Again the Westag 97 plants were tested with the same assay. Westag 97 appeared to encode a functional *Rhg4* resistance allele. Consequently the RLK transgenic Westag 97 showed nearly complet resistance. Therefore, the *GmRLK18-1*-a was alone sufficient to provide for an *Rhg1*–a like activity at different locations in different cultivars. The linked genes may reduce SCN numbers but did require the action of a second locus (*Rhg4*) to provide full resistance to Hg Type 0 as expected from [23,61].

### Analyses of plants transgenic with the resistance allele of the *GmRLK18-1* in the field

Field grown plants showed that the RLK was associated with increased insect herbivory (Table 2C; Figure 5). The primary damaging pest was the Japanese beetle (*Popillia japonica*, Newman). Insects were attracted to the RLK transgenic plants judged by pest incidence (PI) measured from R1-R7 growth stages. Herbivory resulted in plants with less leaf area that produced less shoot biomass at harvest compared to isogenic plants lacking the *GmRLK18*-a allele. There was no incidence of SDS or SCN in the field during the 2010 or 2011 seasons.

### Effect of the Forrest allele of *GmRLK18-1* on transgenic plant development

In both SCN greenhouse assay and field the Forrest allele of *GmRLK18-1* caused a reduction in root and shoot mass (Table 2). The reduction was significant even though the X5 cultivar was innately smaller than the NILs due to a very much earlier maturity date. Reductions occurred in seedlings in the SCN assay (Table 2A), the SDS assay (Table 2B) and whole plants in the field (Table 2C) at harvest maturity. The reduction in growth in the field lead to a reduction in biomass but not seed yield and seed number per plant. Mid- to late-season defoliation by insect pests rarely reduces soybean yield. In both SCN and field assays plant growth was limited by available water. A reduction in root growth might be related to reduced water uptake.

However, in the water saturated assays of SDS resistance the Forrest allele of *GmRLK18-1* caused an increase in root and shoot mass (Table 2) in the presence of *F. virguliforme* infestations. The increase in growth lead to a increase in biomass and seed yield (Figure 5) and seed number per plant in plants grown to maturity in the greenhouse (1.7 ± 0.3 compared to 5.7 ± 1.1). Water sufficiency appeared to make the Forrest allele of *GmRLK18-1* beneficial to growth and yield when *F. virguliforme* was present. In contrast, plants that were not infested in the SDS assays showed the Forrest allele of *GmRLK18-1* caused a decrease in root and shoot mass.

## Discussion

**Figure 6:**
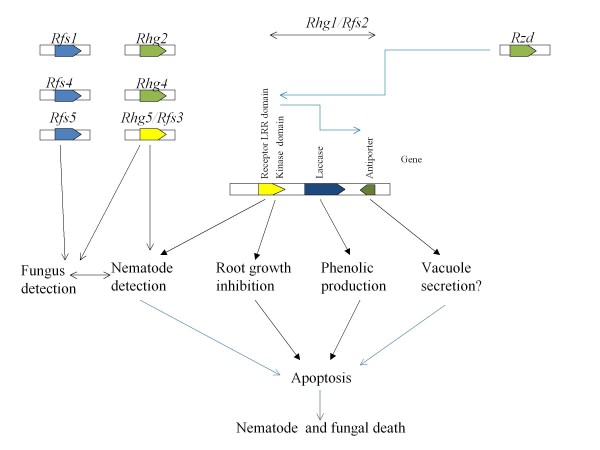
**Genetic model for the function of the *Rhg1/Rfs2* locus in association with other known resistance loci.** Black arrows show positive interactions, blue arrows show inhibitions. In this model four phenotypic events are controlled by the 3 genes at *Rhg1/Rfs2* and four unlinked genes *Rhg2-4* and *Rzd1* (*sup-Rhg1/Rfs2*). Root growth inhibition occurs despite suppression of the RLK by *Rzd1*. Pleiotropy or close linkage between loci underlying resistance was found at *Rhg1/Rfs2* (this work) and *Rhg5/Rfs3 *[8,17].

The *GmRLK18-1* at the *Rhg1/Rfs2* locus was shown to underlie resistance to root infection by *F. virguliforme* and the subsequent leaf scorch, as predicted by [11]. Some resistance to SCN was found but this was partial as expected in the absence of a resistance allele at *Rhg4 *[23,61]. The RLK simultaneously contributed resistance to both pathogens, establishing pleiotropy (Figure 6). Reduced seedling root growth was part of the resistance mechanism and this may underlie the reduced yield of resistant cultivars [39-41]. In Arabidopsis CLAVATA1, the RLK that regulated meristem development, also had an effect on nematode resistance [52].

In field trials of plants transgenic with *GmRLK18-1* further evidence of pleiotropy was discovered when leaf herbivory was shown to be significantly worsened. Insect pests are separated into the chewing and sucking guilds. Resistance mechanisms to the guilds were known to operate by different pathways but some involved the *Rhg4* locus or region [62]. Arabidopsis resistance to *F. graminearum* was recently shown to require operation of both SA and JA pathways [63]. The same Nils used here when infested with H. glycines decreased proteins involved in salicylic acid responses and increased those in the jasmonic acid signaling pathways [46]. Therefore, plants transgenic with Gm18RLK18-1 might be altered in their responses to a broad range of pests and pathogens. Additional pathogens will be tested in future studies.

The *Rhg1/Rfs2* locus was shown to center on three genes where recombination was suppressed within a wider region that was high in recombination. Perhaps the failure to recombine within the central 3 gene cluster caused the unusually high frequency of heterozygous plants in the neighboring region. A potential suppressor locus acting on the resistance allele of *Rhg1* was identified earlier [21,45,47]. The allele of the gene on chromosome 7 (Lg M) had to be inherited from the resistant parent to prevent zygote or embryo lethality in SCN resistant plants. Consequently, all resistant plants co-inherited *Rhg1/Rfs2* on chromosome 18 (Lg G) and the resistance allele on chromosome 7 (Lg M). That gene may have had a functional homeolog in this study. However, the locus was fixed to the R haplotype in all the NILs, so fine mapping was not possible. Plant transgenic with the RLK were fertile and produced seed and they would lack the resistance allele of the gene on chromosome 7 (Lg M). Therefore, *GmRLK18-1* does not cause zygote or embryo lethality. Additional genetic elements must be involved in that phenomenon.

An absence of recombination events in a region can be caused by; deletions; insertions; inversions; the condensed heterochromatin found near centromeres; and recombinant allele lethality. The first four phenomena were not occurring at the *Rhg1/Rfs2* locus since DNA sequences in resistant and susceptible cultivars were co-linear over at least 87 kbp. Further, the *Rhg1/Rfs2* locus mapped into a region within 1 Mbp of the predicted telomere region of chromosome 18. Therefore, recombinant allele lethality may be the cause and a hypothetical model for its action was developed. The kinase domain of the RLK at *Rhg1/Rfs2* was proposed as the element causing lethality to gametes or the zygote carrying a recombination event. The hypothetical anti-porter protein was proposed as the target locus kept in the resistant state when the RLK is conferring SCN resistance (Figure 6). The laccase that was located between these two genes, along with the intergenic regions, are held in phase by the locus. It may be possible that the lethal nature of the three genes linked in association at the resistance locus are not fully suppressed by the modifier locus (they are leaky) and results in the inhibition of root growth observed in SCN resistant cultivars (Figure 2).

The linkage disequilibrium studies reported with probes in, and around, the RLK inferred a larger more complex structure for *Rhg1* than simply the RLK polymorphisms [15,16,25,64]. Possible roles included contributions to additive resistance; contributions to resistance in other resistance types (eg PI88788 and Toyosuzu; R types 2 and 3) or contributions to the resistance to other Hg types. The hypothesis that the linked genes were factors necessary in susceptible genotypes for SCN parasitism was unlikely [16,25,53]. The dominance of *Rhg1/Rfs2* to SCN in NIL segregation also suggested the genes were active in resistant types and inactive in susceptible genotypes.

## Conclusions

The *Rhg1/Rfs2* locus was shown to include the *GmRLK18-1*. Previously [53] also reported inhibition of the resistance allele increased FI in composite roots, but not to a significant degree in small experiments with few replications. Here *Rhg1/Rfs2* was inferred to be a complex of three genes assembled and co-inherited over long periods of selection for resistance to a endemic pest, root parasitic nematodes. However, the *GmRLK18-1* alone at a new genomic locations in transgenic plants was sufficient for resistance to a relatively new pathogen of soybean *F. virguliforme*. Revisions to the sequence of the RLK made by re-sequencing PCR products [25] were confirmed by BAC fragment sequencing here. Fortunately the amino acid changes made to [16] lay outside of regions under detailed analysis for ligand and antibody binding [65,66]. Previously CLE peptide in nematode secretions were shown to be perceived by RLKs in the CLAVATA1, *CLAVATA2* and *CORYNE* families [52]. In vitro, the purified *GmRLK18-1* LRR domain has been shown to bind strongly to the CLE peptide found in nematode secretions as well as two proteins and two CLE peptide of plant origin involved in tracheary element inhibition [43,66]. It will be of interest to discover whether the ligand profiles are similar for the two resistances. The biochemical analysis of the RLK protein will lead to refinements of models for modes of action for the pleiotropic resistance.

The allelic discrimination probes developed provide a high throughput alternative to satellite markers for marker assisted selection. These tools will facilitate molecular breeding for resistance to two or more important pests and diseases of soybean [2]. The discovery of a syntenic set of paralogs to the RLK, laccase and transporter gene cluster at *Rhg1/Rfs2* may also assist marker assisted breeding. The discovery of the syntenic homeologs raised significant barriers to reverse genetic approaches to analyses of the RLK at *Rhg1/Rfs2*. Another barrier to reverse genetic approaches was the role of *Rhg1/Rfs2* in normal plant development that can be inferred from the restricted root growth of NILs (Figure 2) and transgenic plants (Table 2). A third barrier to reverse genetics was the uncoupling in eukaryotes of transcript and protein abundances. Both effects may have contributed to the recent report of no significant difference between the SCN counts on composite plant roots with the GmRLK18-1-b alone targeted for inhibition [53]. It must be noted that the GmRLK18-1-b protein abundance was not measured in that study in spite of the tools available to do so [43,65,66]. Further, note that the numbers of cyst did differ between treatments [53] and that might be significant in larger, replicated studies or under different conditions. Further note that allele was recessive. However, further proofs of the nature of *Rhg1/Rfs2* locus function may require knock-outs of each of the paralogs, or stable transformation to several new locations, followed by measurements of genetic segregation. In each case, the analysis will be complicated by the co-dominant nature of the resistance gene in certain experiments. In fact, the possibility that the susceptible Essex allele of *Rhg1/Rfs2* was functional by promoting root growth and susceptibility, and so actively promoted the establishment of parasitism by SCN and *F. virguliforme*, should be explored with transgenic plants. The model inferring that soybean has adapted part of an existing pathogen recognition and defense cascade (SCN parasitism and insect herbivory), to a new pathogen (*F. virguliforme*) disease (root rot and leaf scorch and syndrome (SDS) has broad implications for crop improvement. Stable resistance to many pathogens might be achieved by manipulation of the genes encoding a small number of pathogen recognition proteins in the RLK family.

## Methods

### Plant materials

Many of the NILs were described previously [7,11,16]. All lines were released and consequently are available on request as seed. Seeds of NIL 34–23 (resistant haplotype between markers Satt 214 to Satt 570) and NIL 34–3 (susceptible haplotype from the marker Satt 214 to the Sat122-Satt 570 interval) were obtained at the F5:13 generation. Genotypes were *rhg1-e/rfs2, rhg1-e/rfs2, Rhg4, Rhg4* for NIL 34–3 and *Rhg1-a/Rfs2, Rhg1-a/Rfs2, Rhg4, Rhg4* for NIL 34–23 whereas NIL34-33 contained both those and *rhg1*-e*/rfs2, Rhg1-*a*/Rfs2, Rhg4, Rhg4* in different plants (NIL34-33-1 to −34).

Soybean cv. X5 and Westag 97 were used for transformation because they could be regenerated to plant efficiently from embryo cultures [67]. They were susceptible to both SCN and SDS. X5 was judged to be *rhg1*-e*/rfs2, rhg1*-e*/rfs2, rhg4, rhg4* and Westag 97 was judged *rhg1-e/rfs2rhg1-e/rfs2, Rhg4, Rhg4* and based on the assays reported here. Crosses were made to RIL EF2 for SCN tests which was *rhg1*-e*/rfs2, rhg1*-e*/rfs2, Rhg4, Rhg4.*

### SCN inoculations

Soybean plants were grown in cones containing a 1:1 ratio of sand soil mix, placed in a water bath to maintain the root zone at 26°C or in a growth chamber at 26°C. Infection with the three different Hg Type 0 SCN populations (PA3, JB3 and YC3) in separate assays consisted of inoculating 2,000 eggs to each 14 day old seedling. Plants were watered daily with 100 ml per pot. Infested soybean plants were removed from the cones at 28 days post infestation (dpi) and cyst numbers counted and compared to ‘Essex’ susceptible controls [16]. Some plant roots were harvested at 10 and 20 days for protein analyses. Root masses of the NILs and transgenic plants were noted. Heterozygous NILs were replanted in non-infested soil and grown to maturity. Seed were harvested. The indicator lines female indices (FI) for nematode population JB3 were ‘PI54840’ (FI 7%), PI 88788 (FI 2%), PI90763 (FI 1%), PI437654 (FI 0%), ‘PI 209332’ (FI 1%), ‘PI89772’ (FI 2%) ‘PI548316’ (FI 8%) and ‘PI548402’ (FI 3%). The soil collected from Yichun in China (YC3) contained Hg Type 0 (SCN race 3) was ‘Peking’ (FI 0%), ‘PI 88788’ (FI 0%), ‘PI 90763’ (FI 6%) and ‘Pickett’ (FI 9%). Essex was the susceptible genotype used across assays to determine 100% FI. Therefore, the standard differentials showed these HG Types to all be variants on Hg Type 0 (Niblack et al. 2003) corresponding to race 3 (Riggs and Schmitt 1988). Nematode population PA3 was described previously [16,23].

### Assays of root development in the absence of infestations

Root development was assayed in NILs and transgenic plants using conditions identical to the SCN assays or SDS assays (as noted in text) except that the roots were not infested with either pathogen. Roots were weighed separately from shoots during destructive sampling. These plant organs were used for RNA and protein extractions.

### SDS measures following *F. virguliforme* infestations

Greenhouse assays of the effects of *F. virguliforme* infestations followed the methods previously described [5]. A culture of *F. virguliforme* virulent strain ‘Mont-1’ (NRRL 22292; MAFF 238545) was provided by Dr. A. Fakhoury (SIUC). Briefly, the strain was grown on ten potato dexrose agar plates. Hyphae and spores were washed with distilled water and 10 μl of this is used for spore count on a hemocytometer under a microscope. Spore counts of 10^4^ spores/cm^3^ of sand and soil mix were used. Seed were sown in sterilized 1:1 (v/v) of sand and soil inoculated with *F. virguliforme* virulent strain Mont-1 in 10 cm square pots. Pots were kept in trays filled to the 5 cm level with water to keep the lower half of the soil saturated. The water was inoculated with 10^4^ spores/cm^3^ to avoid dilution of the inocula in the pots. Transgenic plants, non transgenic X5, selected ExF RILs and a set of breeders advanced lines were grown in the green house at the Southern Illinois University Horticulture Research Center in Carbondale, IL. Experiments were conducted from October 2009 to May 2012. Plants were grown with a 14 h photoperiod under supplemental lights. The air temperature ranged from 20 ± 2°C at night to 27 ± 2°C during the day in the green house. Leaf symptoms were rated every 7 days from infestation to senescence. The standard sudden death syndrome DS score comparable across studies was rated at 21 days after inoculation, determined on the basis of the degree of leaf damage (chlorosis/necrosis) on each plant, and was rated on a scale of 1 to 9 (1 = 0–10%/1–5%, 2 = 10–20%/6–10%, 3 =20–40%/10–20%, 4 =40–60%/20–40%, 5 = 60%/_40% of leaf surface chlorosis/necrosis, respectively, 6 = up to 33% premature defoliation, 7 = up to 66% premature defoliation 8 = 66% premature defoliation, and 9 = premature death of plant). At 28 days after infection (dai) roots were washed, photographed and a root sample (1 g) taken. Root rot severity (RS) was scored on a scale of 1–5 where 1 was unaffected; 2 was discolored; 3 was discolored and partly rotted; 4 was discolored and heavily rotted; and 5 was discolored and necrotic. Plants were weighed, the root to shoot ratio estimated visually and then repotted into the media of the reciprocal genotype to test for pot effects. The experiments were repeated on 3 occasions using 3–5 plants of each genotype. Highly resistant plants of RIL EF23, and highly susceptible plants of RIL EF85 were assayed in parallel with each test.

### DNA and RNA for genotype analysis

DNA was isolated following [11]. Concentrations of DNAs were calculated by measuring absorbances at 260 and 280 nm. Total RNA was isolated with Trizol^TM^ (Invitrogen, Carlsbad, CA, USA), according to the manufacturer’s instructions. First strand cDNA was synthesis carried out using oligo dT primers using a cDNA synthesis kit, according to manufacturer (Invitrogen). Presence of the *Rhg1/Rfs2* resistance alleles was confirmed by PCR analysis using TMD1 an indel marker in the RLK intron. Several designs of TMD1 primers have been reported [11,16,23]. Used here were the primers pair; forward 5′- CAC CTG CAT CAA GAT GAA CA -3′ and reverse 5′- GCC TAT TAC TTG GGA CCC AA -3′ (Additional file 4: Table S2). Genotyping by markers linked to *Rhg1/Rfs2* used about 50 ng of DNA for microsatellite analysis on PAGE after [39] and on agarose gels after [8].

### BAC sequence and allele comparisons

Southern hybridizations were performed following the standard procedure described in [68] to identify paralagous BACs. BACs B73p06 and H38f23 were sequenced at TIGR (nee JCVI). Briefly, the entire BAC was sheared by nebulization to provide fragments in the 3–5 kbp or 9–11 kbp range. The fragments were ligated into pHOS2 and used for Sanger DNA sequencing. BACs were sequenced to 8–12 fold redundancy and assembled. Assembly quality was judged by BLAST comparisions for sequences from A3244 BACs [11,16,50] and Williams 82 genome sequence [54].

### Allelic discrimination at the RLK within the *Rhg1/Rfs2* locus

The SNP genotyping assay within the gene encoding the RLK was performed using a custom Taqman^TM^ Kit. Three probes were designed for the SNPs at 1,486 bp, 506 bp and 2,040 bp (relative to the translation start site) to distinguish the 8 commonest alleles of the RLK (Additional file 4: Table S2). Six process were designed to the non-synonymous SNPs in the GmRLK18-1 preotein to distinguish the seven allotypes. The PCR reactions were carried out using a 3 step PCR protocol with one hold at 95°C for 10 minutes followed by 35 cycles that included a denaturation cycle of 95°C for 30 sec, annealing at 58°C for 10 seconds and an extension at 68°C for 20 sec.

Primers for SNPs within the *Rhg1/Rfs2* locus on Lg G were used in fine melt curve assays as described previously [69] with the following modifications. Briefly, genomic DNA was used; multiple amplicon sizes were detected on PAGE gels; an ABI7900 with HTM software was used; melt curve data were normalized by both local and global metrics. Primers used were to the SNP at 2090–1 for transgenic cDNA between X5 and X5 transgenics. The target was SNP G to T (G for resistant and T for susceptible plants). SNP2090-1 forward primer was 5′- GTT GGT TGA TCC AGA AGG GTT -3′ and the reverse was 5′- CTA AGC TTC CTG AGG CCT TG -3′.

For detection of mRNA products of GmRLK18-1 (Figure 3) the qRT-PCR methods described previously [45-47] were followed. Briefly, mRNA concentrations were estimated from Cot curve analyses using cDNAs.Actin1 was the control used for comparisons.

### Allelic discrimination across the region flanking the *Rfs2/Rhg1* locus

Screens for new recombination events used the flanking markers Satt309 and Satt038. Recombinants identified with those markers were screened with TMD1 and three SNP primers for Taqman^TM^ assays used were set as described in [45-47] (Afzal, 2008b; 2012) with the following modifications. The SNPs used were; AX196295 10893 between the laccase and RLK; AX196295 37583 CR-G in the laccase; and AX196295 37581 CR-G in the hypothetical antiporter gene.

### Total root protein extraction, SDS–PAGE and Western hybridization

Protein from root material was isolated from infested and non-infested roots was extracted after [46]. Total protein concentration was determined using a non-interfering protein assay. For the Western hybridizations, a custom made antibody generated against a peptide [C]TL SRL KTL DIS NNA LNG NLP ATL SNL S from the LRR domain of RLK at RHG1/RFS2 was used (Alpha diagnostics, San Antonio, Texas).

### Transgenic Plants

For soybean transformation, the cassette included pSBHB94 that was a 9.772 kbp insert sub-cloned from BAC B21d09 by nebulization, size fractionated to 9–11 kbp and ligated into pHOS2. The plasmid pSBHB94 encompassed the sequences found from 30,423–40,194 bp in BAC B73p06. Transformation, selection and plant regeneration were conducted after [67]. Briefly, proliferative embryogenic cultures of soybean cv. X5 (AAFC breeding line X2650-7-2-3) or Westag 97 were co-bombarded with the pHOS_SBHB94 and *Hyg*^*R*^[70] constructs; transgenic events were selected and maintained on 55 mg L^−1^ hygromycin; embryos were matured on antibiotic-free medium, air desiccated and converted on B5 medium [71]; tissue cultures and regenerating plantlets were maintained at 20 C and 20 h photoperiod. The plantlets were transferred to soil and plants were regenerated under controlled conditions as in [67]. Primary transgenic (T_0_) plants were tested for the presence of the pHOS::SBHB94 transgene using PCR with the TMD1 primers. Fourteen primary lines were obtained and grown for seed. T_2_ seed from T_1_ plants was tested for transgene segregation to identify homozygous T_1_ individuals. Event 6B3-7D2 and 6B3-7D3 provided seed for the X5 experiments described here. Repeats of the experiments were made on seed of events 8B2-7D2 and 8B2-15D1 in Westag 97.

The genotype of X5 was *rhg1*-e*/rfs2, rhg1*-e*/rfs2, rhg4rhg4.* Purified stable transgenics were of genotype *rhg1*-e*/rfs2rhg1*-e*/rfs2, rhg4rhg4::* GmRLK18-1, GmRLK18-1 (so *Rhg1-*a*/Rfs2Rhg1-*a*/Rfs2*) as shown by markers TMD1 and A2D8*.* Expression of the transgene was established by qRT-PCR from cDNA with allele specific Taqman probes and HRM of amplicons. Protein allotypes were identified by two dimensional PAGE [46] followed by Western hybridization [43,44].

Field trials were conducted at the ARC in Carbondale during 2010 and 2011 using conditions described in [62]. Plants were arrayed in 12′ plots arrayed in a randomized complete block with 4 replications. Insect herbivory was measured as described in [62]. Briefly, the pest incidence was calculated as the number of individual plants within a given line that were affected by herbivorous insects; the pest severity was the percent defoliation; and both were measured once a week from the R1 to R6 growth stages. The major defoliating pest was the Japanese Beetle (*Popillia japonica*, Newman). Plant biomass was measured for 5 plant per plot. The seed yield of each plot was measured after harvest.

## Abbreviations

RLK, Receptor like kinase; SCN, soybean cyst nematode; SDS, sudden death syndrome; BAC, bacterial artificial chromosome; MAFF, Genebank System National Institute of Agrobiological Sciences, Tsukuba, Japan; NRRL, The Agriculture Research Service Culture Collection National Center for Agricultural Utilization Research, USDA/ARS, Peoria, IL USA.

## Competing interest

Authors declare that they have no competing interest.

## Authors’ contributions

AS and AJA contributed equally to the NILs analyses. AS was responsible for the transgenic plant analyses in vitro, DALightfoot for the assays *in vivo*. LB-B and DHS generated all the transgenic plants described here. WL, ML and PA provided SCN assays with YC3 and PA3. NH, and DAL analyszed BAC sequences and generated Figure 1 and Additional file 1: Table S1. CDT provide the BAC sequences. HS carried out the SDS assays on NILs. DAL wrote the manuscript and edited the final version. All authors read and approved the final manuscript.

## Data depositions

TMD1 marker, FJ520231; Corrected RLK at Rhg1/Rfs2 gene AF506516 and mRNA AF506517; SIUC-Satt122, bankit1155667; BAC pB73P06 complete sequence JN597009; pSBHB94 complete sequence HQ008939; BAC pH38f23 complete sequence TBD.

Three supplementary figures and three supplementary tables are published online and links to 7 sequences at Genbank are provided.

## Supplementary Material

Additional file 1: **Figure S1. Paralogs of *Rhg1* in the soybean genome.** Panel (A) shows LRR probe (200bp) hybridized to Forrest MTP. (B) Southern hybridization of LRR probe (200bp) to the MTP positives. Five out of the 7 MTP clones hybridized after BAC clone purification and restriction digestion with HindIII. The lower panel (C) shows the kinase domain probe (200bp) hybridized to Forrest MTP. Panel (D) shows Southern hybridization of the same kinase probe to the MTP positives. Three out of 5 MTP clones hybridized after BAC clone purification and restriction digestion with HindIII. Panel F shows ideograms of the genes predicted from the genome sequences centered on GmRLK18-1 and GmRLK11-1 Panel F shows an alignment of the genome sequences of 70kbp centered on GmRLK18-1 and GmRLK11-1 showing the extent of synteny. Click here for file

Additional file 2: **Table S1. Comparisons of sequence identity between Forrest alleles of GmRLK18-1 and GmRLK11-1 the most similar and syntenic RLK like protein.** The amino acid identity was 78% in the signal peptide (residues 1–61); 94% in the ten LRRs (141–471), 93% in the transmembrane domain (485–507) and 97% in the kinase domain (569–840). Residues that differ in alloproteins of GmRLK18-1 are in bold. Four of the six are identical in the homeoprotein the other 2 are identical to the susceptible allele. The neighboring laccase and the antiporter also showed 85–96% amino acid identity. Click here for file

Additional file 3: **Figure S2. Detection of the SNP polymorphism at position 1486 in the LRR region of *Rhg1* –a and –e using an allelic discriminatory assay.** A Famlabeled probe was used for the detection of resistant haplotypes1 and 2 (red) and Hex labeled probe for the detection of susceptible haplotypes2, 3 and 4 (blue). A total of 16 individuals from the 110 PIs were selected for the analysis. The Panel shows relative fluorescent signal intensity for each of the 16 plant introductions. The two groups form separate clusters. Click here for file

Additional file 4: **Table S2. The sequence of the microsatellite primers that were used for *Rhg1-a* fine map development (from Triwitayakorn et al., 2005).** Click here for file
