# Supplementary material for: The receptor like kinase at Rhg1-a/Rfs2 caused pleiotropic resistance to sudden death syndrome and soybean cyst nematode as a transgene by altering signaling responses
Source: BMC Genomics. 2012 Aug 2;13:368. doi: 10.1186/1471-2164-13-368 (PMC3439264; doi:10.1186/1471-2164-13-368)
Supplement: Additional file 1: — Figure S1. Paralogs of Rhg1 in the soybean genome. Panel (A) shows LRR probe (200bp) hybridized to Forrest MTP. (B) Southern hybridization of LRR probe (200bp) to the MTP positives. Five out of the 7 MTP clones hybridized after BAC clone purification and restriction digestion with HindIII. The lower panel (C) shows the kinase domain probe (200bp) hybridized to Forrest MTP. Panel (D) shows Southern hybridization of the same kinase probe to the MTP positives. Three out of 5 MTP clones hybridized after BAC clone purification and restriction digestion with HindIII. Panel F shows ideograms of the genes predicted from the genome sequences centered on GmRLK18-1 and GmRLK11-1 Panel F shows an alignment of the genome sequences of 70kbp centered on GmRLK18-1 and GmRLK11-1 showing the extent of synteny. [file 1471-2164-13-368-S1.ppt]

## Slide 1
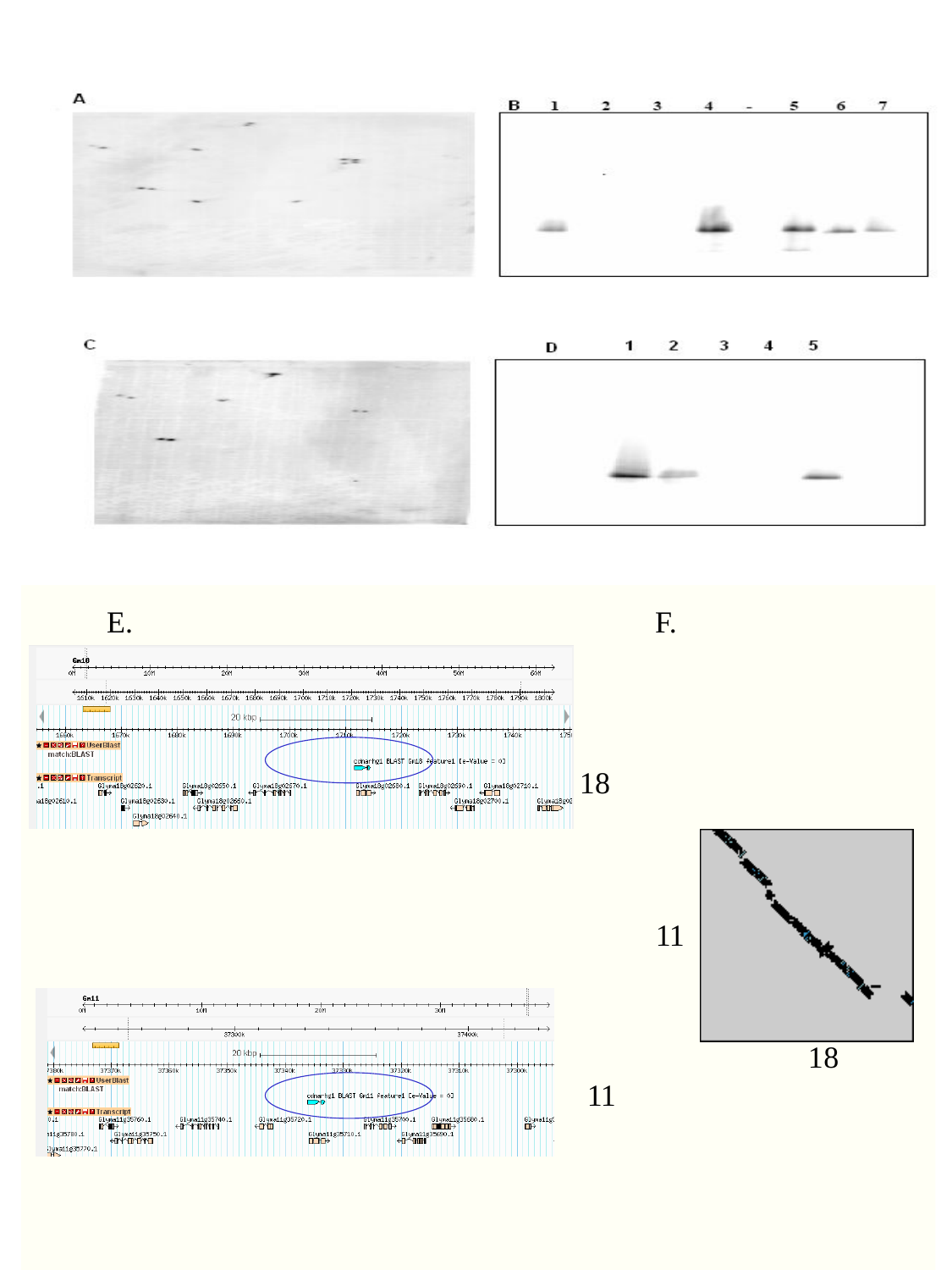

## Slide 2
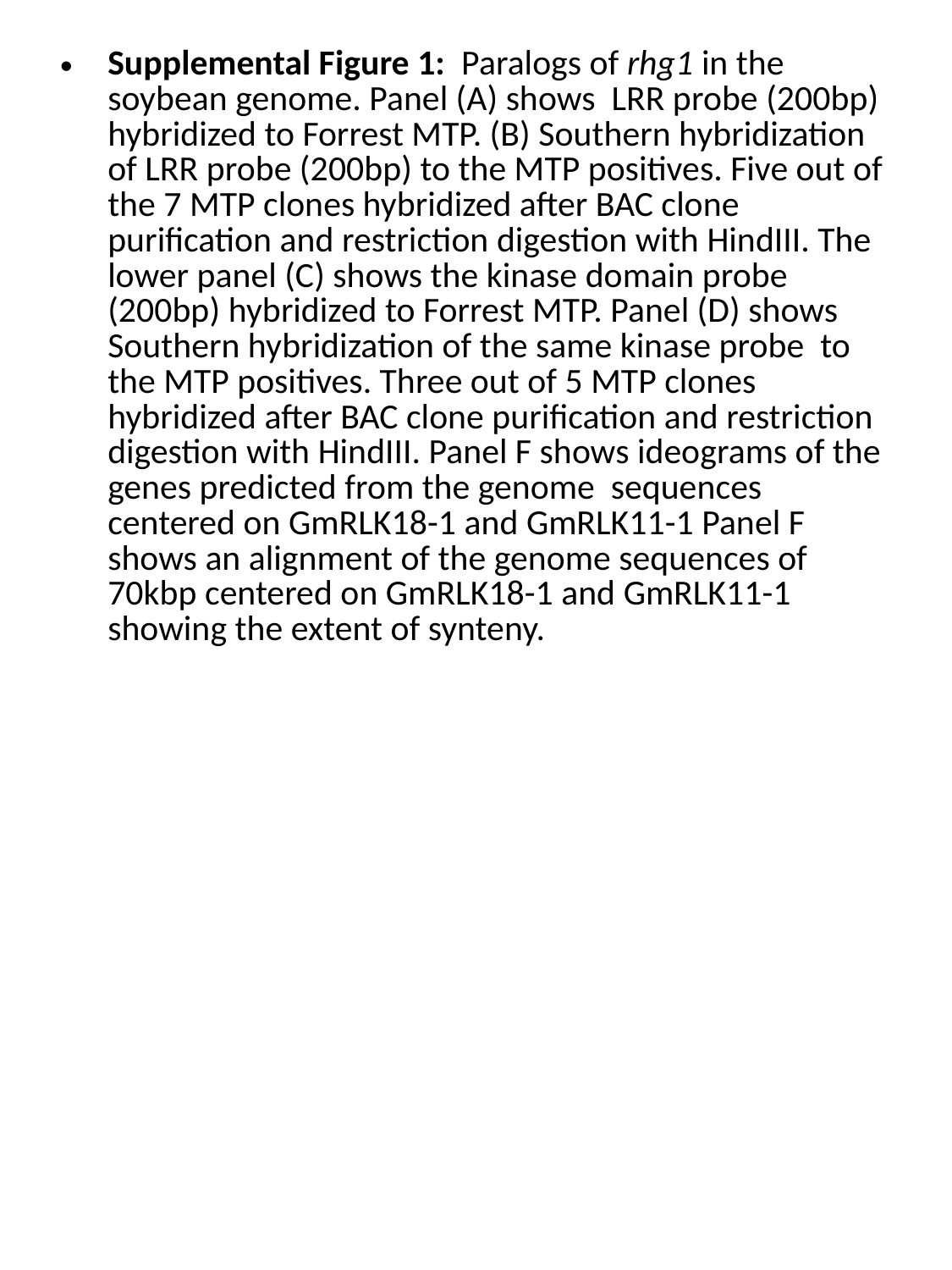

Supplemental Figure 1: Paralogs of rhg1 in the soybean genome. Panel (A) shows LRR probe (200bp) hybridized to Forrest MTP. (B) Southern hybridization of LRR probe (200bp) to the MTP positives. Five out of the 7 MTP clones hybridized after BAC clone purification and restriction digestion with HindIII. The lower panel (C) shows the kinase domain probe (200bp) hybridized to Forrest MTP. Panel (D) shows Southern hybridization of the same kinase probe to the MTP positives. Three out of 5 MTP clones hybridized after BAC clone purification and restriction digestion with HindIII. Panel F shows ideograms of the genes predicted from the genome sequences centered on GmRLK18-1 and GmRLK11-1 Panel F shows an alignment of the genome sequences of 70kbp centered on GmRLK18-1 and GmRLK11-1 showing the extent of synteny.
