# Supplementary material for: The receptor like kinase at Rhg1-a/Rfs2 caused pleiotropic resistance to sudden death syndrome and soybean cyst nematode as a transgene by altering signaling responses
Source: BMC Genomics. 2012 Aug 2;13:368. doi: 10.1186/1471-2164-13-368 (PMC3439264; doi:10.1186/1471-2164-13-368)
Supplement: Additional file 2: — Table S1. Comparisons of sequence identity between Forrest alleles of GmRLK18-1 and GmRLK11-1 the most similar and syntenic RLK like protein. The amino acid identity was 78% in the signal peptide (residues 1–61); 94% in the ten LRRs (141–471), 93% in the transmembrane domain (485–507) and 97% in the kinase domain (569–840). Residues that differ in alloproteins of GmRLK18-1 are in bold. Four of the six are identical in the homeoprotein the other 2 are identical to the susceptible allele. The neighboring laccase and the antiporter also showed 85–96% amino acid identity. [file 1471-2164-13-368-S2.doc]

Supplemental Table 1: Comparisons of sequence identity between Forrest alleles of GmRLK18-1 and GmRLK11-1 the most similar and syntenic RLK like protein. The amino acid identity was 78% in the signal peptide (residues 1–61); 94% in the ten LRRs (141–471), 93% in the transmembrane domain (485–507) and 97% in the kinase domain (569–840). Residues that differ in alloproteins of GmRLK18-1 are in bold. Four of the six are identical in the homeoprotein the other 2 are identical to the susceptible allele. The neighboring laccase and the antiporter also showed 85-96% amino acid identity.

A. GmRLK18-1

>lcl|55921 unnamed protein product RHG1 V RHG_14G5

Length=849 Score = 1572 bits (4070), Expect = 0.0, Method: Compositional matrix adjust.

Identities = 792/854 (93%), Positives = 817/854 (96%), Gaps = 6/854 (1%)

Query 1 MVVAVEKTNLTSQSQCFNRVSDKKKERCKTHMNNVNPCCFLFLLCVWSLVVLPSCVRPVLC 61

++ VEKTNLTSQ CFNR+SDKKKER KTH NN PC LFLLC+WSLVVLPSCVRP LC

Sbjct 1 MLLVEKTNLTSQ--CFNRISDKKKERWKTHNNN--PCRVLFLLCMWSLVVLPSCVRPALC 56

Query 62 EDEGWDGVVVTASNLLALEAFKQEL**A**DPEGFLRSWNDSGYGACSGGWVGIKCA**Q**GQVIVI 121

EDE WDGVVVTASNLLAL+AFKQEL DPEGFLRSWNDSGYGACSGGWVGIKCAQGQVIVI

Sbjct 57 EDESWDGVVVTASNLLALQAFKQELVDPEGFLRSWNDSGYGACSGGWVGIKCAQGQVIVI 116

Query 122 QLPWKGLRGRITDKIGQLQGLRKLSLHDNQIGGSIPSTLGLLPNLRGVQLFNNRLTGSIP 181

QLPWKGL+GRITDKIGQLQGLRKLSLHDNQIGGSIPSTLGLLPNLRGVQLFNNRLTGSIP

Sbjct 117 QLPWKGLKGRITDKIGQLQGLRKLSLHDNQIGGSIPSTLGLLPNLRGVQLFNNRLTGSIP 176

Query 182 LSLGFCPLLQSLDLSNNLLTGAIPYSLANSTKLYWLNLSFNSFSGPLPASLTHSFSLTFL 241

SLGFCPLLQSLDLSNNLLTGAIPYSLANSTKLYWLNLSFNSFSG LP SLTHSFSLTFL

Sbjct 177 SSLGFCPLLQSLDLSNNLLTGAIPYSLANSTKLYWLNLSFNSFSGTLPTSLTHSFSLTFL 236

Query 242 SLQNNNLSGSLPNSWGGNSKNGFFRLQNLILD**H**NFFTGDVPASLGSLRELNEISLSHNKF 301

SLQNNNLSG+LPNSWGG+ K+GFFRLQNLILDHNFFTG+VPASLGSLREL+EISLSHNKF

Sbjct 237 SLQNNNLSGNLPNSWGGSPKSGFFRLQNLILDHNFFTGNVPASLGSLRELSEISLSHNKF 296

Query 302 SGAIPNEIGTLSRLKTLDISNNALNGNLPATLSNLSSLTLLNAENNLLDNQIPQSLGRLR 361

SGAIPNEIGTLSRLKTLDISNNA NG+LP TLSNLSSLTLLNAENNLL+NQIP+SLG LR

Sbjct 297 SGAIPNEIGTLSRLKTLDISNNAFNGSLPVTLSNLSSLTLLNAENNLLENQIPESLGTLR 356

Query 362 NLSVLILSRNQFSGHIPSSIANISSLRQLDLSLNNFSGEIPVSFDSQRSLNLFNVSYNSL 421

NLSVLILSRNQFSGHIPSSIANIS LRQLDLSLNN SGEIPVSF+SQRSL+ FNVSYNSL

Sbjct 357 NLSVLILSRNQFSGHIPSSIANISMLRQLDLSLNNLSGEIPVSFESQRSLDFFNVSYNSL 416

Query 422 SGSVPPLLAKKFNSSSFVGNIQLCGYSPSTPCLSQAPSQGVIAPPPEV-SKHHHHRKLST 480

SGSVPPLLAKKFNSSSFVGNIQLCGYSPSTPCLSQAPSQGVIAP PEV S+ HH R LST

Sbjct 417 SGSVPPLLAKKFNSSSFVGNIQLCGYSPSTPCLSQAPSQGVIAPTPEVLSEQHHRRNLST 476

Query 481 KDIILIVAGVLLVVLIILCCVLLFCLIRKRSTSKAGNGQATEGRAATMRTEKGVPPVA**G**G 540

KDIILIVAGVLLVVLIILCC+LLFCLIRKRSTSKA NGQAT GRAAT RTEKGVPPV+ G

Sbjct 477 KDIILIVAGVLLVVLIILCCILLFCLIRKRSTSKAENGQAT-GRAATGRTEKGVPPVSAG 535

Query 541 DVEAGGEAGGKLVHFDGPMAFTADDLLCATAEIMGKSTYGTVYKAILEDGSQVAVKRLRE 600

DVEAGGEAGGKLVHFDGP+AFTADDLLCATAEIMGKSTYGTVYKAILEDGSQVAVKRLRE

Sbjct 536 DVEAGGEAGGKLVHFDGPLAFTADDLLCATAEIMGKSTYGTVYKAILEDGSQVAVKRLRE 595

Query 601 KITKGHREFESEVSVLGKIRHPNVLALRAYYLGPKGEKLLVFDYMSKGSLASFLHGGGTE 660

KITKGHREFESEVSVLGK+RHPNVLALRAYYLGPKGEKLLVFDYM KG LASFLHGGGTE

Sbjct 596 KITKGHREFESEVSVLGKVRHPNVLALRAYYLGPKGEKLLVFDYMPKGGLASFLHGGGTE 655

Query 661 TFIDWPTRMKIAQDLARGLFCLHSQENIIHGNLTSSNVLLDENTNAKIADFGLSRLMSTA 720

TFIDWPTRMKIAQD+ RGLFCLHS ENIIHGNLTSSNVLLDENTNAKIADFGLSRLMSTA

Sbjct 656 TFIDWPTRMKIAQDMTRGLFCLHSLENIIHGNLTSSNVLLDENTNAKIADFGLSRLMSTA 715

Query 721 ANSNVIATAGALGYRAPELSKLKKANTKTDIYSLGVILLELLTRKSPGV**S**MNGLDLPQWV 780

ANSNVIATAGALGYRAPELSKLKKANTKTDIYSLGVILLELLTRKSPGVSMNGLDLPQWV

Sbjct 716 ANSNVIATAGALGYRAPELSKLKKANTKTDIYSLGVILLELLTRKSPGVSMNGLDLPQWV 775

Query 781 ASVVKEEWTNEVFDADLMRDASTVGDELLNTLKLALHCVDPSPSARPEVHQVLQQLEEIR 840

AS+VKEEWTNEVFDAD+MRDASTVGDELLNTLKLALHCVDPSPS RPEVHQVLQQLEEIR

Sbjct 776 ASIVKEEWTNEVFDADMMRDASTVGDELLNTLKLALHCVDPSPSVRPEVHQVLQQLEEIR 835

Query 841 PERSVTASPGDDIV 854

PERSVTASPGDD +

Sbjct 836 PERSVTASPGDDTI 849

B. Gmlaccase18-1 partial

>lcl|731 unnamed protein product LACCASE RHG1 V PART OF LACCASE ON 14G5

Length=179

Sort alignments for this subject sequence by:

E value [Score](http://blast.ncbi.nlm.nih.gov/Blast.cgi?CMD=Get&ALIGNMENTS=100&ALIGNMENT_VIEW=Pairwise&BLAST_SPEC=blast2seq&DATABASE_SORT=0&DESCRIPTIONS=100&DISPLAY_SORT=0&FIRST_QUERY_NUM=0&FORMAT_OBJECT=Alignment&FORMAT_PAGE_TARGET=&FORMAT_TYPE=HTML&GET_SEQUENCE=yes&I_THRESH=&MASK_CHAR=2&MASK_COLOR=1&NEW_DESIGN=on&NEW_VIEW=yes&NUM_OVERVIEW=100&OLD_BLAST=false&PAGE=Proteins&QUERY_INDEX=0&QUERY_NUMBER=0&RESULTS_PAGE_TARGET=&RID=TR6Y7NW8114&SHOW_LINKOUT=yes&SHOW_OVERVIEW=yes&STEP_NUMBER=&WORD_SIZE=3&HSP_SORT=1#731) [Percent identity](http://blast.ncbi.nlm.nih.gov/Blast.cgi?CMD=Get&ALIGNMENTS=100&ALIGNMENT_VIEW=Pairwise&BLAST_SPEC=blast2seq&DATABASE_SORT=0&DESCRIPTIONS=100&DISPLAY_SORT=0&FIRST_QUERY_NUM=0&FORMAT_OBJECT=Alignment&FORMAT_PAGE_TARGET=&FORMAT_TYPE=HTML&GET_SEQUENCE=yes&I_THRESH=&MASK_CHAR=2&MASK_COLOR=1&NEW_DESIGN=on&NEW_VIEW=yes&NUM_OVERVIEW=100&OLD_BLAST=false&PAGE=Proteins&QUERY_INDEX=0&QUERY_NUMBER=0&RESULTS_PAGE_TARGET=&RID=TR6Y7NW8114&SHOW_LINKOUT=yes&SHOW_OVERVIEW=yes&STEP_NUMBER=&WORD_SIZE=3&HSP_SORT=3#731)

[Query start position](http://blast.ncbi.nlm.nih.gov/Blast.cgi?CMD=Get&ALIGNMENTS=100&ALIGNMENT_VIEW=Pairwise&BLAST_SPEC=blast2seq&DATABASE_SORT=0&DESCRIPTIONS=100&DISPLAY_SORT=0&FIRST_QUERY_NUM=0&FORMAT_OBJECT=Alignment&FORMAT_PAGE_TARGET=&FORMAT_TYPE=HTML&GET_SEQUENCE=yes&I_THRESH=&MASK_CHAR=2&MASK_COLOR=1&NEW_DESIGN=on&NEW_VIEW=yes&NUM_OVERVIEW=100&OLD_BLAST=false&PAGE=Proteins&QUERY_INDEX=0&QUERY_NUMBER=0&RESULTS_PAGE_TARGET=&RID=TR6Y7NW8114&SHOW_LINKOUT=yes&SHOW_OVERVIEW=yes&STEP_NUMBER=&WORD_SIZE=3&HSP_SORT=2#731) [Subject start position](http://blast.ncbi.nlm.nih.gov/Blast.cgi?CMD=Get&ALIGNMENTS=100&ALIGNMENT_VIEW=Pairwise&BLAST_SPEC=blast2seq&DATABASE_SORT=0&DESCRIPTIONS=100&DISPLAY_SORT=0&FIRST_QUERY_NUM=0&FORMAT_OBJECT=Alignment&FORMAT_PAGE_TARGET=&FORMAT_TYPE=HTML&GET_SEQUENCE=yes&I_THRESH=&MASK_CHAR=2&MASK_COLOR=1&NEW_DESIGN=on&NEW_VIEW=yes&NUM_OVERVIEW=100&OLD_BLAST=false&PAGE=Proteins&QUERY_INDEX=0&QUERY_NUMBER=0&RESULTS_PAGE_TARGET=&RID=TR6Y7NW8114&SHOW_LINKOUT=yes&SHOW_OVERVIEW=yes&STEP_NUMBER=&WORD_SIZE=3&HSP_SORT=4#731)

Score = 322 bits (824), Expect = 1e-92, Method: Compositional matrix adjust.

Identities = 162/190 (85%), Positives = 164/190 (86%), Gaps = 19/190 (10%)

Query 1 MEPAKTIHNNVKYSPIFL--AIFVLILASALSSANAKIHEHEFVVEATPVKRLCKTHNSI 58

ME K I+ N K+S IFL IFVLILASA NAKIHEHEFVVEATPVKRLCKTHNSI

Sbjct 1 MESVKFINMNAKHSSIFLLAMIFVLILASA----NAKIHEHEFVVEATPVKRLCKTHNSI 56

Query 59 TVNGQYPGPTLEINNGDTLVVKVTNKARYNVTIHWYNIKLASMAFFSGHGVRQMRTGWAD 118

TVNGQYPGPTLEINNGDTLVVKVTNKARYNVTIHW HGVRQMRTGWAD

Sbjct 57 TVNGQYPGPTLEINNGDTLVVKVTNKARYNVTIHW-------------HGVRQMRTGWAD 103

Query 119 GPEFVTQCPIRPGGSYTYRFTVQGQEGTLWWHAHSSWLRATVYGALIIRPREGEPYPFPK 178

GPEFVTQCPIRPGGSYTYRFTVQGQEGTLWWHAHSSWLRATVYGALIIRPREGEPYPFPK

Sbjct 104 GPEFVTQCPIRPGGSYTYRFTVQGQEGTLWWHAHSSWLRATVYGALIIRPREGEPYPFPK 163

Query 179 PKHETPILLG 188

PKHETPILLG

Sbjct 164 PKHETPILLG 173
