# Supplementary material for: The receptor like kinase at Rhg1-a/Rfs2 caused pleiotropic resistance to sudden death syndrome and soybean cyst nematode as a transgene by altering signaling responses
Source: BMC Genomics. 2012 Aug 2;13:368. doi: 10.1186/1471-2164-13-368 (PMC3439264; doi:10.1186/1471-2164-13-368)
Supplement: Additional file 3: — Figure S2. Detection of the SNP polymorphism at position 1486 in the LRR region of Rhg1 –a and –e using an allelic discriminatory assay. A Famlabeled probe was used for the detection of resistant haplotypes1 and 2 (red) and Hex labeled probe for the detection of susceptible haplotypes2, 3 and 4 (blue). A total of 16 individuals from the 110 PIs were selected for the analysis. The Panel shows relative fluorescent signal intensity for each of the 16 plant introductions. The two groups form separate clusters. [file 1471-2164-13-368-S3.pdf]

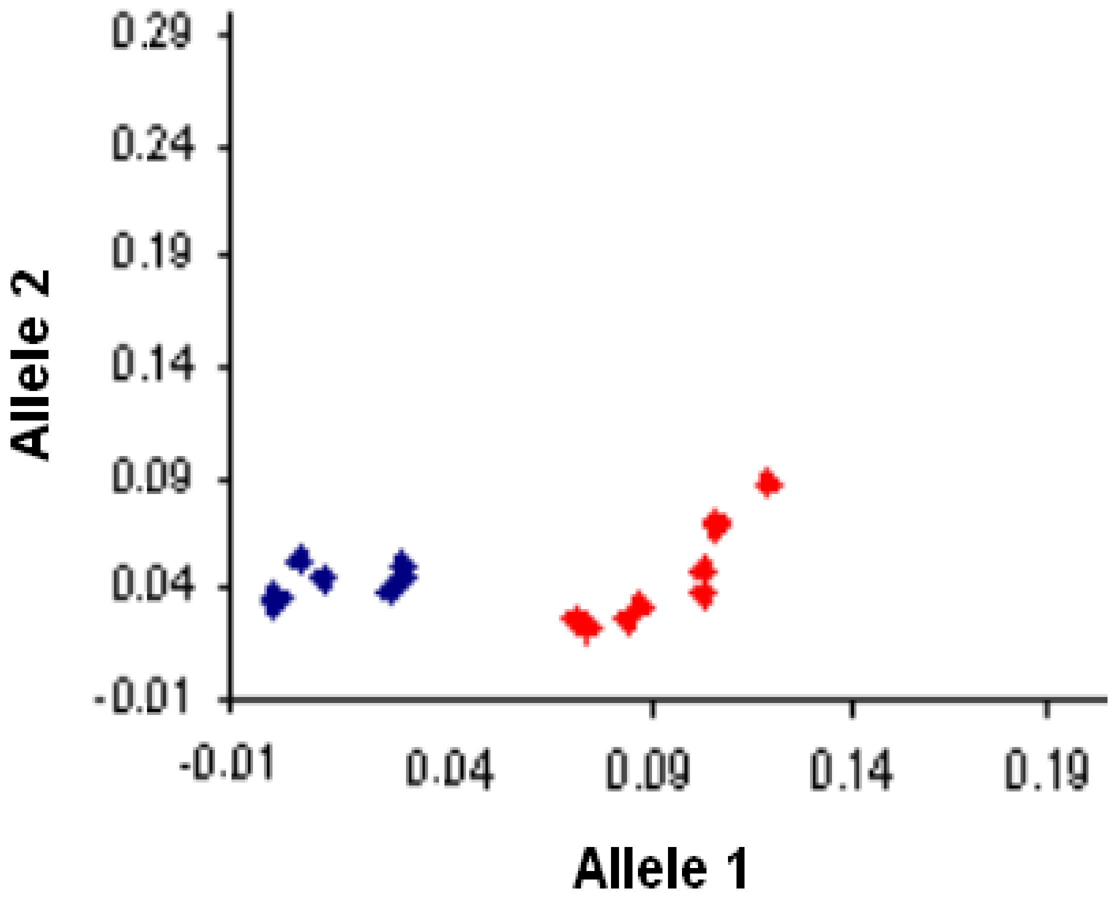
0.29
0.24
0.19
0.14
0.09
0.04
-0.01
Allele 2
-0.01
0.04
0.09
0.14
0.19
Allele 1

- **Supplemental Figure 2:** Detection of the SNP polymorphism at position 1486 in the LRR region of *Rhg1* –a and –e using an allelic discriminatory assay. A Fam labeled probe was used for the detection of resistant haplotypes 1 and 2 (red) and Hex labeled probe for the detection of susceptible haplotypes 2, 3 and 4 (blue). A total of 16 individuals from the 110 PIs were selected for the analysis. The Panel shows relative fluorescent signal intensity for each of the 16 plant introductions. The two groups form separate clusters.
