# Supplementary material for: The receptor like kinase at Rhg1-a/Rfs2 caused pleiotropic resistance to sudden death syndrome and soybean cyst nematode as a transgene by altering signaling responses
Source: BMC Genomics. 2012 Aug 2;13:368. doi: 10.1186/1471-2164-13-368 (PMC3439264; doi:10.1186/1471-2164-13-368)
Supplement: Additional file 4: — Table S2. The sequence of the microsatellite primers that were used for Rhg1-a fine map development (from Triwitayakorn et al., 2005). [file 1471-2164-13-368-S4.doc]

Supplementary Table 2A: The sequence of the microsatellite primers that were used for *rhg1* fine map development (from Triwitayakorn et al., 2005).

| Primer | Sequence (5’ – 3’); R shown as reverse complement | | Tm^a^  (^o^C) | AmpliconGenBank# | Coordinates in DNA Sequence AX196295 (and BAC73p06) | Reference |
| --- | --- | --- | --- | --- | --- | --- |
| BARC-Satt309 | F | GCG CCT TCA AAT TGG CGT CTT | 64 | BH126500 | NP^a^ 71361^b^ (60843) | Song et al., 2004 |
|  | R | GCG CCT TAA ATA AAA CCC GAA ACT | 68 |  | NP 71501 ^b^ (61217) |  |
| SIUC-Sat_122 | F | CTC ACA AAA TTG AAA TGT ATC | 54 | AR370653 | NP | Meksem *et al*., 2000 |
|  | R | CCT TTT TCA TCT TCA AAA T | 48 |  | NP |  |
| SIUC-Sat_001 | F | TAC TAT ATG ACC CAA CAG | 50 | AY858582 | 34634 (24296) | Ruben et al.,2006^d^ |
|  | R | CTA ATG CTA AGT GTC ACG | 50 |  | 34857 (24657) |  |
| BARC-Satt570 | F | CTC ATG TGG TCC TAC CCA GAC TCA | 74 | BH126736 | NP | Song et al., 2004 |
|  | R | CGC TAT CCC TTT GTA TTT TCT TTT GC | 72 |  | NP |  |
| SIUC-Satt127 (Satt128) | F | ATG GGT TTT GGG GAT TTC AC | 55 | AY858579 | NP: 97317 in AX196294^C^ | Triwitayakorn et al., 2005^d^ |
|  | R | AGA AGA GGA TGC TAA GTT GC | 53 |  | NP; 97729 in AX196294^C^ |  |
| SIUC-Sat_185,(Sat138) | F | CAG AGT CAA GTT TGA ACTT CAT | 60 | AY858580 | 185494 | Ruben et al 2006^d^ |
|  | R | AAT CAA GTG ATC ATT GTA CTG | 56 |  | 185911 |  |
| SIUC-Sat_075 (Sat_027) | F | CAT TTA ACC TTA CTA ATC TAA A | 54 | AY858575 | 75801 (65544) | Ruben et al., 2006^d^ |
|  | R | CAT GTA TTA AAT AAT TTA TGC | 50 |  | 76181 (65904) |  |

a NP signifies the exact match of the primer sequence is not present.

b a paralog of the expected sequence was present. c is a second sequence tract of 127 kbp.

d Primers were identified and used in 2000-2001, independently of Hauge et al., 2001.

Supplementary Table 2B: The sequence of the microsatellite primers that were used for fine map development by Ruben et al. 2006.

| Primer | Sequence (5’ – 3’; R not reverse complement) | | Tm^a^  (^o^C) | AmpliconGenBank# | Coordinates in DNA Sequence AX19629 (and BAC73p06) | Reference |
| --- | --- | --- | --- | --- | --- | --- |
| SIUC-Sat027 | F | ATTTAACCTTACTAATCTAAA | 64 | AY858575 | 75801 (65544) | Ruben et al. 2006 |
|  | R | GCATAAATTATTTAATACATG | 68 |  | 76181 (65904) |  |
| SIUC-Sat037 | F | ATGCACATTTTGGAGCCATTC | 54 | AY858574 | 84371 (74005) | Ruben et al. 2006 |
|  | R | ATGCTAGGGCCTAATTTGAA | 48 |  | 84691(74440) |  |
| SIUC- Sat046 | F | ATACATAAGTCATCCGATGAA | 50 | AY858576 | 93131 | Ruben et al. 2006 |
|  | R | AATACACTCTCAATGCATAAC | 50 |  | 93431 |  |
| SIUC-Sat069 | F | AACTAAATCCTCCAAACA | 74 | AY858577 | 116821 | Ruben et al. 2006 |
|  | R | AGAAAAGAGAATTGGCAAAGT | 72 |  | 117191 |  |
| SIUC-Satt096 | F | TGTTACTTAGTAATTATGAAG | 55 | AY858578 | 143492 | Ruben et al. 2006 |
|  | R | ATCGATCAACAAATCATTATT | 53 |  | 143811 |  |
| SIUC-Sat128 | F | TGGTTCATGTAATTTTATCAT | 60 | AY858579 | 175921 | Ruben et al. 2006 |
|  | R | TTTTTAGTCTCTATACTTTGA | 56 |  | 176332 |  |
| SIUC-Sat138 | F | CAGAGTCAAGTTTGAACTTCAT | 54 | AY858580 | 185494 | Ruben et al. 2006 |
|  | R | CAGTACAATGATCACTTGATT | 50 |  | 185911 |  |

a Primers were identified and used in 2000-2001, independently of Hauge et al., 2001.

Supplementary Table 2: C. The sequence of the SCAR primers that were used for *rhg1* mapping.

| Primer | Sequence (5’ – 3’) | | Tm^a^  (^o^C) | Amplicon GenBank # | Coordinates in Sequence AX196295 | Reference |
| --- | --- | --- | --- | --- | --- | --- |
| ATG4-SCAR^b^  EATGMCGA87 | F | CTG TGG ATT GAA TTC ATG GT | 56 | AF489439 | 84422 (74135) | Meksem *et al*., 2001 |
|  | R | TAT TAA CGA CCA CGG TTA TC | 56 |  | 84519 (74221) |  |
| CTA13-SCAR  ECTAMAGG113 | F | GAA TTC CTA ATA TAC GAG | 48 | AF489440 | 152205 | Meksem *et al*., 2001 |
|  | R | AGG GAT ATG TTT TTT TCA C | 50 |  | 152318 |  |
| SattTMD1  Int1Kin^c^ | F | AAA CTA GTC CAT TTT GAT GGA | 56 | AY858573 | 48448 (38061) | Triwitayakorn et al., 2005 |
|  | R | AGC ATT TGT ATT CTC ATC AAG | 56 |  | 49126 (38803) |  |
| TMD1-indel | F | GAG AGC CTA TTA CTT GGG ACC C | 56 | AF506516 | 48699 (38311) | This Work |
|  | R | CCA CCA CCT GCA TCA AGA TGA AC | 54 |  | 49002 (38679) |  |
| CGG5-SCAR ECGGMAGA116 | F | CAC GAT TAT CCA GGC TTT GAGAGG | 72 | AF489441 | 228732 | Meksem *et al*., 2001 |
|  | R | AGG AAT AGA AGA CAG TGT CAG GA | 70 |  | 228816 |  |
| SIUC-SAC13 SIUC-Sca05^c^ | F | CAAGTATGCTTCATGAATAGTTAC | 66 | AY858583 | 46479 (36101) | Ruben et al., 2006 |
|  | R | GATGCAAGACACACATGAACAACG | 62 |  | 46900 (36641) |  |

^a^ Tm (^o^C) = 4(G+C) + 2(A+T)

b paralogs of the expected sequence were also present

c Primers were identified and used in 2000-2001, independently of Hauge, et al., 2001.

Supplementary Table 2D: The sequence of the markers and primers that were developed from BAC73p06 sequences and used for fine map development

| Primer | Sequence (5’ – 3’; R) | | Tm^a^  (^o^C) | AmpliconGenBank# | Coordinates in DNA Sequence of BAC73p06 and AX19629 ) | Reference |
| --- | --- | --- | --- | --- | --- | --- |
| SIUC_Sat_-36 | F | TGTCCCTAAATAAATTAATAAATCCAA | 56 | TBD | 1,770 (11,870) | This work |
|  | R | AAATGACCCTCTCTCTCTCT | 58 |  |  |  |
| *SIUC_Scaa-23* | F | To be released on publication | 54 | TBD | 13,800 (23,900) | This work |
|  | R | To be released on publication | 48 |  |  |  |
| Minisatt 1  (InD-3.5) | F | AGACAAAGCATTCTGATCGC | 59 | TBD | 33,900 (44,000) | This work |
|  | R | AGATTCGCCACTACTGTTGG | 60 |  |  |  |
| SIUC_Satt3.5 | F | CCCAACATAATTCCAACTTCA | 58 | TBD | 40,150 (50,000) | This work |
|  | R | GCAACAATGCTAGCCATCAA | 59 |  |  |  |
| SIUC-Saaag3.5 | F | TTCCAACTTCAAAATTCACTCAA | 57 | TBD | 40,200 (50,050) | This work |
|  | R | GTGTCATCAACAACGCAACA | 60 |  |  |  |
| SIUC-Satt9.0 | F | GCACTAATGGTGACACACAC | 59 | TBD | 45,250 (54,750) | This work |
|  | R | TGAGTTAGACCTCCTCTCTTGT | 60 |  |  |  |
| SIUC-Satt9.5 | F | CTTTAAAGTCTCAAGTCATTGGAT | 57 | TBD | 45,500 (55,000) | This work |
|  | R | CGAAAGGGCCTCTTTATGTT | 58 |  |  |  |
| SIUC-Satt22 | F | CCTTGCAATAGAGGAGTACAA | 57 | TBD | 58,500 (58,500) | This work |
|  | R | AGTACGTGTCTTGATTTTATTTCTT | 57 |  |  |  |
| SIUC Satt39 | F | GTTTTGGCAAGCAAGAGTCC | 60 | TBD | 75,800 (85,900) | This work |
|  | R | AACTAGTATGGTATAAAGTAAGGCT | 57 |  |  |  |
| SIUC-Sat_40 | F | GCATGTTTGGCTCGCATTAAA | 60 | TBD | 79,500 (89,600) | This work |
|  | R | TCCCACTTCAATTAACTCATGC | 59 |  |  |  |

Supplemental Table 2E: SNP Primers from Hyten et al 2007

  SNP gi# LG bp Forward primer                          Reverse primer                   Tm
 AX196295 10893 CR-G 54040 GCCCCCCATTTGTCTTGCGTG GCGTGCCGTGGAATCAAAAGTTAG 60
 AX196295 37583 CR-G 62107 GCATTGTCACTCCTGAGAACC ACCGCATCATCACCACCTC 64
 AX196295 37581 CR-G 64929 CCTCTTCTCATCACCGCATTG CAGCACCATTCGCAACCTC 60
 AX196295 37585 CR-G 73698 TTCCAGCGGCGAGACTAAC GCGACTTCACGAGGCAATG 63

Supplemental Table 2 F: Taqman markers for SNPs in the RLK at Rhg1

Rhg1 RLK residue 87

AHMSIJ4_F CAGCATCAAACCTCTTAGCACTTG 36

AHMSIJ4_R CATTCCAGCTCCGCAAGAAC 36

AHMSIJ4_V VIC CTGGATCAGCCAACTC 8 NFQ

AHMSIJ4_M FAM TCTGGATCAACCAACTC 8 NFQ

Rhg1 RLK residue 71

AHKAL7R_F CCAGTTTTGTGTGAAGATGAAGGTT 36

AHKAL7R_R GCTTCAAGTGCTAAGAGGTTTGATG 36

AHKAL7R_V VIC CTGTCACAACCACTCC 8 NFQ

AHKAL7R_M FAM CTGTCACAGCCACTCC 8 NFQ

Rhg1 RLK residue 115

AHLJKDZ_F CGGAGGTTGGGTTGGAATCAA 36

AHLJKDZ_R CCCTCAAACCCTTCCAAGGAA 36

AHLJKDZ_V VIC CTGTCCCTGAGCACAC 8 NFQ

AHLJKDZ_M FAM CCTGTCCCTTAGCACAC 8 NFQ

Rhg1 RLK residue 274

AHQJC2V_F TGGGAATTCCAAGAATGGCTTCT 36

AHQJC2V_R GCAGGAACGTCACCAGTGAAA 36

AHQJC2V_V VIC TTGATCCTAGATCATAACTT 8 NFQ

AHQJC2V_M FAM TTTGATCCTAGATAATAACTT 8 NFQ

Rhg1 RLK residue 539

AHT97LJ_F CGGCCACTATGAGGACAGAAAA 36

AHT97LJ_R CCTCCCCACCTGCTTCAAC 36

AHT97LJ_V VIC CAGTTGCTGGTGGTGAT 8 NFQ

AHT97LJ_M FAM CAGTTGCTGTTGGTGAT 8 NFQ

Rhg1 RLK residue 770

AH0IYGN_F CTACAGTCTTGGTGTTATCTTGTTAGAACT 36

AH0IYGN_R GCAACCCACTGAGGCAAATCTA 36

AH0IYGN_V VIC CCATTCATAGACACCCC 8 NFQ

AH0IYGN_M FAM CCATTCATAGGCACCCC 8 NFQ

Rhg1 RLK base pair 560

RLK560_F CAGTGAAACTTGGAGATGCCAG 57

RLK560_R TCAGTCTCAAATTAGTGAGGGAT 54.7

RLK560_Hex-BHQ1 AAGTGTGATACACACTATCCCTCCTCCTCGT 65.1

RLK_560_Fam-BHQ1 AAGTGTGATACATACTATCCCTCCTCCTCGTGC 65.3
